# Characterization of Endogenous Human FcγRIII by Mass Spectrometry Reveals Site, Allele and Sequence Specific Glycosylation*[Fn FN1]

**DOI:** 10.1074/mcp.RA118.001142

**Published:** 2018-12-17

**Authors:** Nathaniel Washburn, Robin Meccariello, Jay Duffner, Kristen Getchell, Kimberly Holte, Thomas Prod'homme, Karunya Srinivasan, Robert Prenovitz, Jonathan Lansing, Ishan Capila, Ganesh Kaundinya, Anthony M. Manning, Carlos J. Bosques

**Affiliations:** From the ‡Research Department, Momenta Pharmaceuticals, 301 Binney St., Cambridge, Massachusetts 02142

**Keywords:** Mass Spectrometry, Glycoproteomics, Glycoprotein Structure*, N-Glycosylation, Immunology*, Fc-gamma receptors, Neutrophil, Polymorphisms

## Abstract

The importance of IgG glycosylation, Fc-gamma receptor (FcγR) single nucleotide polymorphisms and FcγR copy number variations in fine tuning the immune response has been well established. There is a growing appreciation of the importance of glycosylation of FcγRs in modulating the FcγR-IgG interaction based on the association between the glycosylation of recombinant FcγRs and the kinetics and affinity of the FcγR-IgG interaction. Although glycosylation of recombinant FcγRs has been recently characterized, limited knowledge exists on the glycosylation of endogenous human FcγRs. In order to improve the structural understanding of FcγRs expressed on human cells we characterized the site specific glycosylation of native human FcγRIII from neutrophils of 50 healthy donors and from matched plasma for 43 of these individuals. Through this analysis we have confirmed site specific glycosylation patterns previously reported for soluble FcγRIII from a single donor, identified FcγRIIIb specific Asn45 glycosylation and an allelic effect on glycosylation at Asn162 of FcγRIIIb. Identification of FcγRIIIb specific glycosylation allows for assignment of FcγRIIIb alleles and relative copy number of the two alleles where DNA/RNA is not available. Intriguingly the types of structures found to be elevated at Asn162 in the NA2 allele have been shown to destabilize the Fc:FcγRIII interaction resulting in a faster dissociation rate. These differences in glycosylation may in part explain the differential activity reported for the two alleles which have similar *in vitro* affinity for IgG.

## Abstract (precis)

Characterization of endogenous FcγRIII glycosylation from healthy donors with different FcγRIIIb genotypes reveals site specific, and allele specific differences in glycosylation as well as noncananonical sequence specific differences in glycosylation. We propose these differences in glycosylation may influence the differential activity seen for neutrophils across genotypes.

## Graphical Abstract

**Figure:**
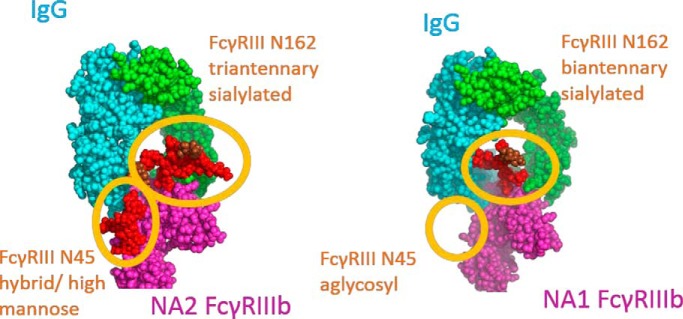

**Highlights**

- Glycosylation of endogenous FcγRIII from neutrophils and matched plasma from more than 40 donors characterized at two sites involved in IgG binding.
- Glycosylation of soluble FcγRIII glycosylation at N45 can be used to assign FcγRIIIb alleles.
- FcγRIIIb allele specific differences in glycosylation at N162 may influence differential activity observed for primary cells.

Receptors for the Fc region of IgG (FcγRs)^1^ are critical in modulating the adaptive immune response. Interaction between the receptors and IgG in immune complexes or on opsonized cells promotes downstream effector function such as antibody dependent cellular phagocytosis (ADCP) and antibody dependent cellular cytotoxicity (ADCC). In humans, there are five activating FcγRs specifically FcγRI (CD64), FcγRIIa (CD32A), FcγRIIc (CD32c), FcγRIIIa (CD16A), and FcγRIIIb (CD16B) as well as the inhibitory FcγRIIb (CD32B) (1). FcγRIIIa and FcγRIIIb are two closely related proteins with at least 95% homology in the amino acid sequence of the extracellular domains which are nearly indistinguishable when considering the common variants (Fig. 1).

**Fig. 1.**
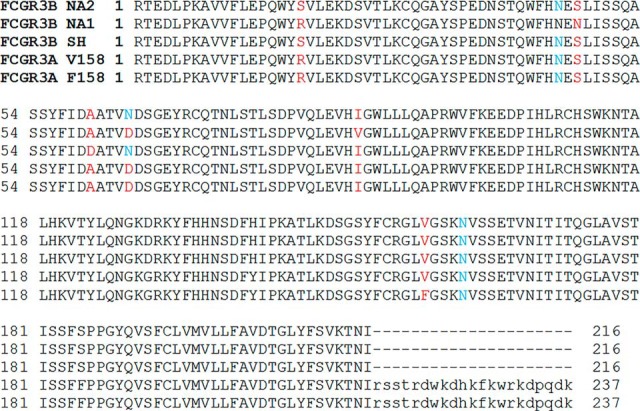
**Alignment of human FcγRIII variants.** Sites of glycosylation that were analyzed are noted in blue; sites defining the FcγRIIIb variants and FcγRIIIa functional V158F variant are noted in red. Sequences for the mature protein were aligned excluding the signal peptide.

FcγRIIIa is expressed on NK cells, and subsets of monocytes, macrophages and dendritic cells. The cytoplasmic domain of FcγRIIIa associates with the immunoreceptor tyrosine-based activation motif (ITAM) containing common FcRγ chain which drives intracellular signaling events (2). The V158F polymorphism which is found in the extracellular domain of FcγRIIIa results in increased affinity between the V158 variant and all IgG subclasses (3). Functionally, NK cells bearing FcγRIIIa with the V158 variant exhibit enhanced response to immune complex stimulation (4). FcγRIIIb is a glycophosphatidylinositol (GPI) linked protein expressed primarily on neutrophils and basophils (2).

FcγRIIIb is highly polymorphic with three common alleles differing at 5 sites in the protein (Fig. 1). Alleles named NA1, NA2 and SH or alternately HNA-1a, HNA-1b and HNA-1c have been described (2), (5) and additional variants have been detected (6). These variants do not influence the affinity of the FcγR-IgG interaction (3) but have been reported to influence neutrophil activity (7), (8), and (9). Both FcγRIIIa and FcγRIIIb are heavily glycosylated. FcγRIIIa contains five potential sites of glycosylation at N38, N45, N74, N162 and N169. The NA1 and NA2/SH alleles are distinguished by amino acid differences at four sites specifically R19S, N47S, D65N and V89I for the NA1 and NA2 alleles respectively. The SH allele is distinguished from the NA2 alleles by A61D substitution (5). The NA2 allele of FcγRIIIb is potentially glycosylated at the five sites found in FcγRIIIa and has an additional consensus site at N65. The NA1 allele on the other hand has only four potential sites of glycosylation because of allelic variation. The N45 and N65 sites are not glycosylated in the NA1 allele because of the presence of N47 and D65 respectively.

Glycosylation has long been established as a critical parameter influencing the FcγR-IgG interaction with core fucosylation (10) and sialylation (11) of the Fc domain of IgG being the best studied. FcγRIII glycosylation has additionally been reported to play a role in the Fc:FcγRIII interaction. Point mutations targeting each of the *N*-linked glycans from FcγRIII demonstrated that the glycans at N45 and N162 play a role in the formation of the Fc:FcγRIII complex. Removal of the N45 glycosylation site increased the affinity of the interaction between the Fc bearing nonfucosylated glycans and FcγRIIIa (12). A separate study using a similar approach demonstrated that the N-glycans at N162 were required for the higher affinity interaction seen for nonfucosylated glycans on the Fc (13). The crystal structure of the complex between FcγRIIIa bearing high mannose type glycans (14) or neutral complex glycans (15) and the Fc domain for human IgG1 showed the interaction was stabilized by carbohydrate-protein and carbohydrate-carbohydrate interactions primarily involving FcγRIII glycans at N162. A recent study utilizing NMR to characterize the solution phase dynamics of glycoengineered FcγRIIIa expressed in HEK cells identified unexpected contacts between the glycans at N45 and the polypeptide backbone (16).

Initial studies examining influence of FcγR glycosylation on the FcγRIII:Fc interaction relied on mutagenesis to selectively remove entire glycan chains. Subsequent studies utilized recombinant proteins produced in different cell types to examine the influence of the nature of the glycans present on the receptor on Fc:FcγRIII binding. Several groups have published data on recombinant FcγR glycosylation from BHK (17), NS0 (18), CHO and HEK (19), (20). The FcγRIII glycosylation pattern varied significantly among expression systems and was demonstrated to influence the kinetics but not the affinity of the interaction when comparing CHO and HEK expressed protein (19). A recent publication compared the effect of expression system on glycosylation pattern of recombinant FcγRI and FcγRIIIa and reproduced the differential kinetics reported by Zeck *et al.* (20). The authors proposed that glycosylation differences, principally branching and sialylation, destabilized the interaction resulting in more rapid dissociation of the complex. These studies complement those utilizing point mutations and provide detail on the influence of FcγR glycan structure on the Fc-FcγR interaction. The advances in LC-MS based characterization of glycopeptides in the past decade (21), provide a means for monitoring site specific glycosylation changes (22) of proteins from complex biological systems (23). Characterization of site-specific glycosylation patterns of endogenous human FcγRs can help to advance an understanding of the impact of FcγR glycosylation on immune cell activation.

Here we present the characterization of native FcγRIIIb glycosylation from isolated human neutrophils as well as soluble FcγRIII, which is a mixture of FcγRIIIa and FcγRIIIb, isolated from matched plasma. Through this analysis we identified FcγRIIIb specific glycosylation at N45 and an allelic influence on glycosylation of N162, which are consistent with and expand upon recent reports.

## EXPERIMENTAL PROCEDURES

### 

#### 

##### Healthy Donor Samples

Matched plasma and neutrophils were obtained from healthy donors after informed consent through a combination of an internal blood donor program as well as a commercial source (Sanguine Bio; Sherman Oaks CA). The collection, handling and biomolecular analysis of healthy human neutrophils per experimental protocol 102013–001 was approved by the Western Institutional Review Board. Plasma was collected in EDTA tubes.

##### Neutrophil Isolation

Neutrophils were isolated from lysed whole blood by negative selection using the Neutrophil Enrichment Kit (StemCell Technologies, Vancouver, BC, Canada; Catalogue #19257) following manufacturer's instructions. Freshly isolated neutrophils were pelleted and frozen at −80 °C until they were used for analysis. Paired neutrophils and plasma from a patient were from the same draw.

##### Multiplexed Ligation-dependent Probe Amplification

For 33 of these donors FcγR polymorphisms and copy number were determined using Multiplexed Ligation-dependent Probe Amplification (MLPA). MLPA assays were performed using commercially available kits from MRC-Holland (P110–100R and P111–100R) using methods similar to those previously described (24), (25). This kit contains probes for determining copy number variation (CNV) and single nucleotide polymorphisms (SNPs) in the FCGR2/C locus. Genomic DNA was extracted from neutrophils from healthy donors using the QIAamp DNA Mini Blood Kit (QIAgen, Hilden, Germany). DNA was denatured at 95 °C and incubated with MLPA probes at 60 °C for a minimum of 18 h. Ligation and PCR amplification were performed according to MLPA manufacturer instructions. Fragment analysis was performed on an ABI-3730XL capillary electrophoresis instrument using POP7 polymer. Data were analyzed using the Coffalyser software (MRC-Holland) according to software manufacturer instructions. FCGR3B allele types and copy number were determined using probes 06639-L06203, 03616-L02983, and 03616-L02990; copy number determination was confirmed by FCGR3B allele-independent probes 03618-L02985 and 03615-L12809.

##### Isolation of Neutrophil Fc Gamma Receptors

Neutrophil FcγRIIIb was isolated from ∼5 million neutrophils. Plasma FcγRIII was isolated from 50 μl of plasma. Proteins were immunoprecipitated using biotinylated goat polyclonal antibodies against human FcγRIII (R&D Systems, Minneapolis, MN; BAF1597). The proteins were isolated from neutrophils by first spinning down cells at 300 × *g* for 2 min and then washing 3 × 500 μl of ice cold PBS. Then 75 μl of IP Lysis Buffer (ThermoFisher Scientific, Waltham, MA; 87787) was added to each sample and cells were lysed by sonication and cell debris spun out at 10,000 × *g* for 5 min. PBS was added to the supernatant to bring the volume to 500 μl then the biotinylated antibody was added and allowed to incubate for 18 h at 4 °C. The antibody-FcγR complex was isolated using streptavidin magnetic beads (ThermoFisher Scientific 88816). The beads were washed two times 500 μl of IP Lysis buffer and two times with 500 μl of ice-cold PBS. The bound protein was eluted by incubating the beads in 50 μl of 6 m guanidine HCl. The eluted protein was reduced for 30 min at 65 °C with DTT at a concentration of 25 mm. Free cysteine residues were alkylated with iodoacetamide at a concentration of 75 mm. The isolated proteins were dialyzed across a 10kDa membrane against 4L of 25 mm ammonium bicarbonate for 18 h at 4 °C before proteolysis. Soluble FcγRs were isolated from 50 μl of plasma as described above omitting the cell lysis and centrifugation step.

##### Proteolysis, Exoglycosidase Treatment and nLC-MS/MS Analysis

The glycosylation pattern of FcγRIII N45 was characterized using a chymotrypsin (Sequencing Grade Promega, Madison, WI; V1061) which cleaves C-terminal to hydrophobic residues such as tyrosine, tryptophan and phenylalanine to generate the glycopeptides shown in Table I. A sequential digestion with endoproteinase GluC (Sequencing Grade Promega V1651) which cleaves C-terminal to glutamic acid followed by chymotrypsin was used to characterize the N162 glycopeptides. We have focused on site specific glycosylation at N45 and N162 because removal of these two sites of glycosylation was demonstrated to influence the interaction between FcγRIII and IgG. The glycopeptide at N45 is common to FcγRIIIa and the NA2 and SH alleles of FcγRIIIb whereas the NA1 allele is nonglycosylated at this site. The glycopeptide at N162 is common to FcγRIIIa and all alleles of FcγRIIIb (Table I).

**Table I:** Species-specific peptide sequences generated from proteolysis of FcγRIII

Sequence	Species	Variant
FID**A(61)**ATV**D(65)**DSGEY	FcγRIIIb NA1/FcγRIIIa	FcγRIII A61/D65
FID**A(61)**ATV**N(65)**DSGEY	FcγRIIIb NA2	FcγRIII A61/N65
FID**D(61)**ATV**N(65)**DSGEY	FcγRIIIb SH	FcγRIII D61/N65
FH**N(45)**E**S(47)**LISSQASSY	FcγRIIIb NA2/SH/FcγRIIIa	FcγRIII S47/N45 glycopeptides
FH**N(45)**E**N(47)**LISSQASSY	FcγRIIIb NA1	FcγRIII N47
SDPVQLEVH**I(89)**GW	FcγRIIIb NA2/SH/FcγRIIIa	FcγRIII I89
SDPVQLEVH**V(89)**GW	FcγRIIIb NA1	FcγRIII V89
VGSK**N(162)**VSSE	FcγRIII N162	FcγRIII N162 glycopeptides

The peptides and glycopeptides were analyzed by nLC-MS/MS on a Dionex Ultimate 3000 nano RSLC (ThermoFisher Scientific) coupled to a QExactive mass spectrometer (ThermoFisher Scientific) equipped with and EasySpray nano-LC source (ThermoFisher Scientific). Peptides were separated on an EasySpray C18 column (0.75 × 250 mm 2 μm particle size ThermoFisher Scientific ES802). A data dependent acquisition was run initially to identify glycopeptides from each site. Glycopeptides were identified by searching the high-resolution accurate mass MS/MS spectra for Y1 ions which tend to form readily under HCD fragmentation of N-glycopeptides. Nonreducing end oxonium ions diagnostic for a variety of structural features including N-acetyllactosamine extensions and antennary fucose including Lewis and sialyl-Lewis structures were used to differentiate isomeric species. The structures of selected isomeric glycans were confirmed using low energy CID fragmentation on an Orbitrap Velos (ThermoFisher Scientific). Glycopeptide fragmentation for structural characterization was visualized using GlycoWorkbench (26).

Isomers arising from differences in sialic acid linkages were characterized using sialidase S according to manufacturer's instruction. This enzyme preferentially cleaves α2–3 linked sialic acid under the conditions used. Multiple chromatographic peaks were identified for each of the sialylated species across all sites.

##### Targeted nLC-MS/MS for Donor Characterization

After initial identification a targeted Tier 3 nLC-MS/MS method was applied for the quantitation of site specific glycosylation as well as assignment of allelic variants based on peptide sequence information. The quadrupole isolation width was set to ±1.5 Da for the isolation of the parent ion of each of the species for the chymotryptic digest (Table II) and the chymotryptic+GluC digest (Table III). The AGC was on the QExactive was set to 1,000,0000 ions and a normalized collision energy of 27 was used for glycopeptide fragmentation. Targeted species were confirmed based on the full MS/MS and quantified based on the extracted ion abundance for the most abundant fragment. Glycopeptides fragmented by HCD give rise to a characteristic Y1 ion which when detected with HR/AM provides a marker fragment unique to each glycopeptide backbone. Identification of the glycopeptides was dependent upon the presence of the Y1 ion in the MS/MS spectrum as well as the presence of at least two specific peptide fragments and the characteristic oxonium ions (supplemental Fig. S2, supplemental Fig. S3). The relative abundance of each glycopeptide was determined using the extracted ion abundance of the Y1 ion relative to the summed extracted ion abundance of this fragment from all glycopeptides at each site of glycosylation. The specific precursor and fragment ions monitored for each species are shown in supplemental information (supplemental Fig. S2).

**Table II:** Characterization of alleleic variants and N45 glycosylation. Species monitored for targeted MS/MS analysis of FcγRIII chymotryptic digest. Isolation width was set at 3 Da. The species monitored are described in the comments column. The species with glycan compositions represent glycopeptide while those with the FcγRIIIb alleles are peptide specific markers for those alleles

Mass [m/z]	Start [min]	End [min]	(N)CE	Species
**701.3**	42	50	25	FcγRIII N65D NA1/NA2
**798.9**	35	41	25	FcγRIII N47 NA1
**723.8**	30	60	25	FcγRIII D61 SH
**683.3**	47	53	25	FcγRIII V89 NA1
**690.5**	50	56	25	FcγRIII I89 NA2
**1394.1**	32	38	25	FcγRIIIb S47 N45 M5
**1475.1**	32	38	25	FcγRIIIb S47 N45 M6
**1556.1**	31	37	25	FcγRIIIb S47 N45 M7
**1094.8**	36	42	25	FcγRIII S47 N45 M4A1G1S1
**1143.5**	36	42	25	FcγRIII S47 N45 FM4A1G1S1
**1637.1**	31	37	25	FcγRIIIb S47 N45 M8
**1718.2**	30	37	25	FcγRIIIb S47 N45 M9
**1148.8**	36	42	25	FcγRIII S47 N45 M5A1G1S1
**1040.0**	36	42	25	FcγRIII S47 N45 A1G1S1
**1088.8**	36	42	25	FcγRIII S47 N45 FA1G1S1

**Table III:** Characterization of N162 glycosylation. Species monitored for targeted MS/MS analysis of FcγRIII GluC+chymotrypsin digest. Isolation width was set at 3 Da

Mass [m/z]	Composition
**1038.8**	N162 FA2G2S1F1
**1111.4**	N162 FA3G3S1
**1160.1**	N162 FA3G3S1F1
**1209.2**	N162 FA3G3S2
**868.0**	N162 FA1G1S1
**989.7**	N162 FA2G2S1
**892.7**	N162 FA2G2
**1086.7**	N162 FA2G2S2
**1135.4**	N162 FA2G2S2F1
**1257.2**	N162 FA3G3S2F1
**941.5**	N162 FA2G2F1
**1306.2**	N162 FA3G3S3
**453.7**	N162 Agycosyl

##### Data Analysis for Identification of Alleleic Variants and Relative Quantitation of Site-specific Glycosylation

Data analysis was performed using Xcalibur software Qual browser. Each glycopeptide species was quantified based on the MS/MS extracted ion current (XIC) for the ion corresponding to the Peptide+N-acetylglucosamine fragment. The relative abundance was calculated by dividing the XIC area of each species by the summed XIC area for each site. FcγRIIIb alleles were identified based on the presence of marker peptides and specific glycosylation at N45; the specific markers used at each site for the assignment the FcγRIIIb alleles are shown below (Table IV). For assignment of alleles and comparison to copy numbers the abundance of N45 high mannose type glycopeptides was calculated relative to the sum of all high mannose glycopeptides plus the NA1 specific aglycosyl peptide. For presentation of N-glycopeptide distribution the aglycosyl NA1 peptide was not included in the calculation. In order to estimate site occupancy at N65 from the NA2 allele the area of the nonglycosylated peptide was normalized to the area of the D65 peptide from the NA1 allele for heterozygotes. Similarly, the area of the NA2 specific I89 peptide was normalized to the NA1 specific V89 peptide. The ratio of these normalized areas was calculated for donors having both alleles. Because the normalized area N65 to D65 but not the normalized area of I89 to V90 is affected by the presence of glycosylation at N65 a ratio close to 1 is indicative of low site occupancy and ratios ≪1 indicative of relatively high site occupancy.

##### Experimental Design and Statistical Rationale

The genotypes of the subjects included in this study were 29% (*n* = 14) NA1/NA1, 35% (*n* = 17) NA1/NA2 and 35% (*n* = 17) NA2/NA2 and 4% (*n* = 2) NA1/SH genotype. FcyR glycosylation from neutrophils and plasma was analyzed one time for each donor. Statistical comparisons between the alleles were performed using unpaired two-sided *t* test. Associations between glycoforms and copy number variants were assessed using one-way ordinary ANOVA. *p* < 0.05 was considered significant.

## RESULTS

### 

#### 

##### Characterization of Neutrophil FcγRIIIb Alleles

The FcγRIIIb alleles for 50 donors were determined from FcγRIIIb protein isolated from neutrophils *via* LC-MS/MS. The alleles were assigned based on the presence of allele specific peptide sequences including the glycopeptide containing N45 (Table IV). Using this approach several donors with noncanonical sequences were identified. Because of the variability observed in the protein the decision was made to genotype the FCGR3B gene in a subset of these donors using Multiplexed Ligand-dependent Probe Amplification (MLPA). This technique provides genetic information as well as information about copy number variants which have been reported to be common for FcγRIIIb (27). Our MLPA data shows that copy number variants are common for FcγRIIIb consistent with recent reports. Assignment of alleles was more than 95% concordant between the two methods with one of the noncanonical donors analyzed by both methods being the only divergent result out of the 22 donors (supplemental Table S1).

**Table IV:** Marker peptides and glycopeptide sequences for assignment of FcγRIII alleles

*Fc*γ*RIIIb NA1*	*Fc*γ*RIIIb NA2*	*Fc*γ*RIIIb SH*	*Fc*γ*RIIIa*
FHN(45)EN(47)LISSQASSY Aglycosylated	FHN(45)ES(47)LISSQASSY Glycosylated	FHN(45)ES(47)LISSQASSY Glycosylated	FHN(45)ES(47)LISSQASSY Glycosylated
FIDAA(61)TVD(65)DSGEY	FIDA(61)ATVN(65)DSGEY	FIDD(61)ATVN(65)DSGEY	FIDAA(61)TVD(65)DSGEY
SDPVQLEVH**V(89)**GW	SDPVQLEVH**I(89)**GW	SDPVQLEVH**I(89)**GW	SDPVQLEVH**I(89)**GW

Assignment of FcγRIIIb allele based on the protein sequence and N45 glycosylation identified two types of noncanonical donors. Two donors appeared to be homozygous for the NA1 allele based on the absence of the N65 peptide and N45/S47 glycopeptide. However, these donors had both the NA1 V89 the NA2 I89 variant suggesting they have one canonical NA1 allele and one noncanonical NA1 (supplemental Fig. S9, S10, and S11). Two other donors appeared to be homozygous for the NA2 allele based on the absence of the NA1 specific aglycosyl N45/N47 and V89 peptides. However, these donors lacked the NA2 specific N65 peptide and instead had the NA1 D65 variant. Interestingly, the noncanonical NA2/NA2 donors also had lower levels of high mannose type glycans at N45 suggesting a possible influence of N65 on the glycosylation at N45 (see Fig. 4*E*, 4*F*).

##### Overview of FcγRIIIb Site Specific Glycosylation

**Fig. 2.**
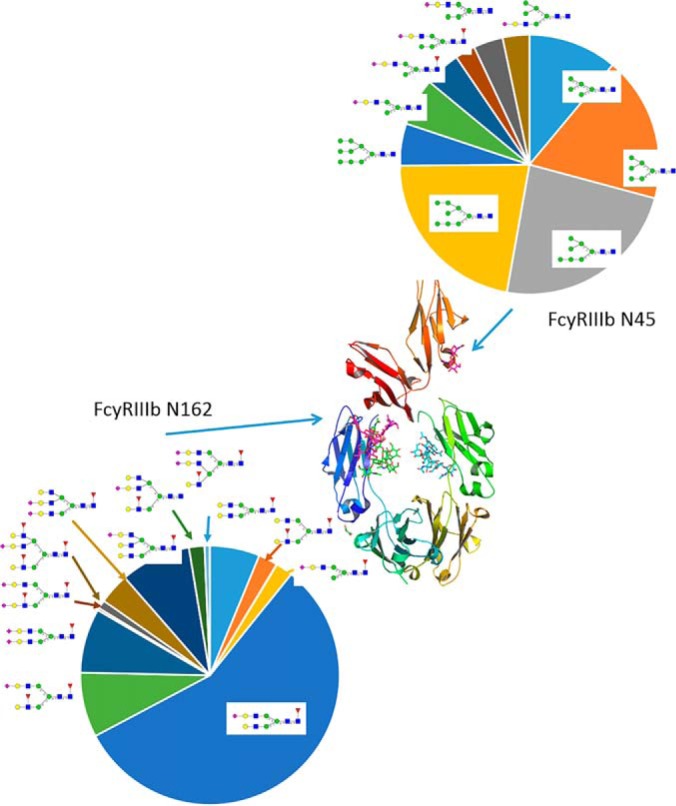
**FcγRIII site specific glycan structures with average abundance across the 50 healthy donors.** Crystal structure for FcγRIII (3SGK (14)) is also shown to illustrate the position of the glycosylation sites relative to the FcγRIII:Fc binding site.

Site specific glycosylation was characterized for N45 and N162 two of the sites common to both FcγRIIIb and FcγRIIIa. These were selected because glycosylation of these two sites has been demonstrated to influence the interaction between FcγRIII and the Fc domain of IgG (13), (12). Consistent with recent reports (28) distinct site-specific glycosylation patterns were identified for these two sites with almost no overlap between species identified at the two (Fig. 2).

**Fig. 3.**
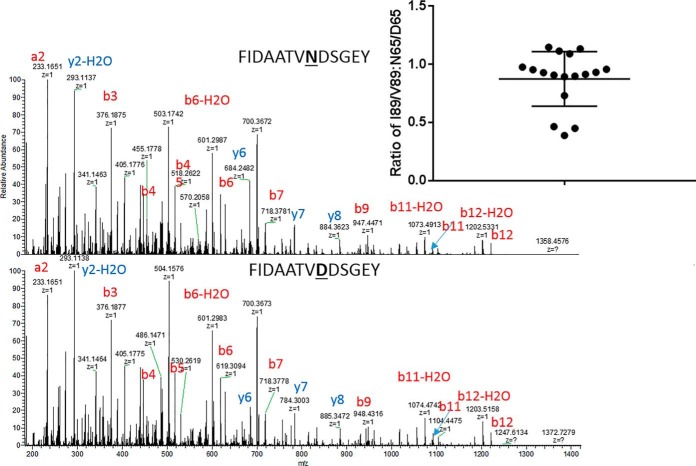
**MS/MS fragmentation for the unoccupied N65 glycopeptide from NA2 compared with the nonglycopeptide D65 from NA1.** The area of the NA1 specific V89 peptide was normalized to the NA2 specific I89 peptide and the NA1 specific D65 peptide was normalized to the NA2 specific N65 and the ratio of the normalized area was taken as described in the methods section. The inset graph shows distribution of this ratio for NA1/NA2 heterozygotes.

The NA2 allele of FcγRIIIb contains two additional potential sites of glycosylation compared with the NA1 allele with N45 and N65 are specific to NA2 allele. N65 was recently reported to be glycosylated in native FcγRIII from human serum (28) we detected only the nonglycoyslated N65 peptide in donors having at least one canonical NA2 allele. Comparing the ratio of marker peptides for the different alleles across the 20 heterozygous donors suggests the NA2 specific site at N65 is largely unoccupied with only 3 of the 17 heterozygous donors having a ratio ≪1 when analyzing FcγRIIIb from isolated neutrophils (Fig. 3). Although N45 was found to be fully occupied based on the absence of the nonglycosylated N45/S47 peptide.

##### N-glycans at N45 Are High Mannose and Sialylated Hybrid Type

Glycopeptides from FcγRIII N45 were characterized using a chymotryptic digest which generated a single major glycopeptide backbone with the sequence FHN(45)ES(47)LISSQASSY. Ten unique FcγRIIIb N45 glycopeptide masses were identified which eluted as 15 distinct chromatographic peaks. The N-glycans identified at FcγRIII N45 are high mannose type and sialylated hybrid type glycans (Table V). The sialylated glycans were found to contain both α2–6 and α2–3 linked sialic acid with the former being more abundant (Table V). No nonglycosylated N45 peptide was detected in FcγRIII protein with the consensus NxS/T motif as is the case for FcγRIIIa and the NA2 allele of FcγRIIIb suggesting this site is fully occupied in the NA2 allele and FcγRIIIa. These results are largely consistent with recent reports which identified high mannose type glycans at this site from FcγRIII isolated from serum (28).

**Table V:**
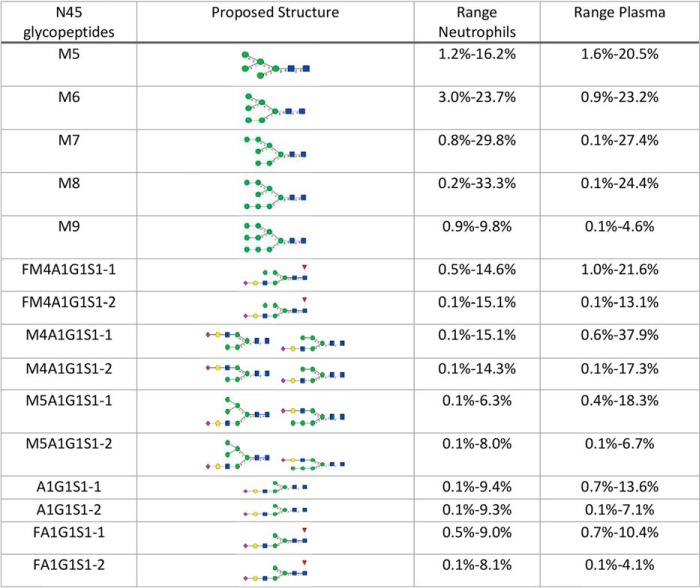
N45 N-glycan levels from FcγRIIIb isolated from neutrophils of donors with at least one NA2 allele and from N45 from FcγRIIIa/b isolated from the plasma of all donors. Oxford nomenclature used for N45 glycopeptides column. Glycan structures were generated based on CFG guidelines using GlycoWorkBench. Multiple potential structures are shown when more detailed structural information could not be obtained

##### FcγRIIIb Contains High Mannose and Sialylated Hybrid N-glycans Whereas FcγRIIIa Contains Only Sialylated Hybrid N-glycans

**Fig. 4.**
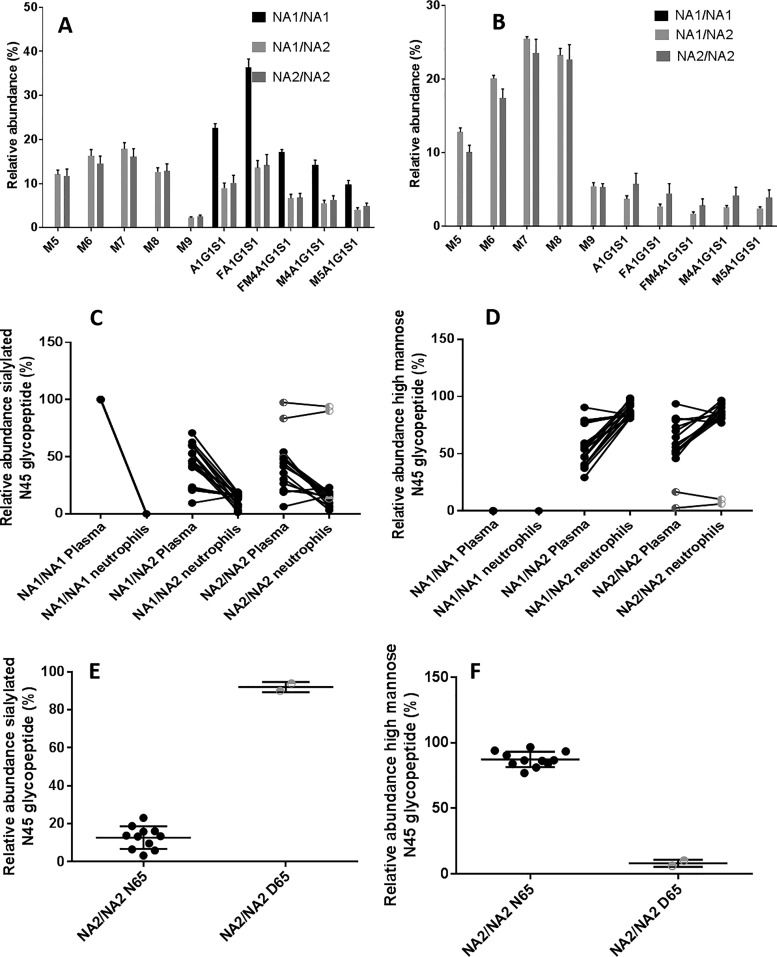
**N-glycopeptide distribution at N45 from plasma (*A*) and isolated neutrophils (*B*) for donors with different FcγRIIIb alleles.** Comparison of the abundance of sialylated (*C*) or high mannose type N-glycans (*D*) at N45 relative to all N45 glycoforms from neutrophils and from plasma. Comparison of the levels of sialylated (*E*) and high mannose type (*F*) N-glycans for NA2/NA2 homozygotes with canonical N65 and noncanonical D65 The aglycosyl NA1 peptide is excluded from this calculation. Mean values shown with S.E. for NA1/NA1 *n* = 9 for plasma and paired, *n* = 14 for neutrophils, NA1/NA2 *n* = 18 for plasma and paired, NA2/NA2 *n* = 14 for plasma and paired, *n* = 19 for neutrophils. The standard error is calculated for each allele based on a single replicate for each donor within the group.

Comparing the glycosylation patterns between matched plasma and neutrophils from healthy donors suggests high mannose type glycans at N45 are specific to FcγRIIIb. FcγRIII isolated from the plasma of donors homozygous for the NA1 allele show only hybrid sialylated structures (Fig. 4*A*). The lack of the consensus glycosylation motif in FcγRIIIb means that N45 glycans of plasma FcγRIII from donors homozygous for the NA1 allele must be contributed entirely by FcγRIIIa. FcγRIII isolated from plasma of donors with at least one NA2 allele contains a mixture of high mannose and hybrid structures (Fig. 4*B*). The lack of high mannose type glycans in both plasma and neutrophils from NA1 homozygotes as well as the presence of these species in donors with at least one NA2 allele suggests high mannose type glycans at N45 can be used as a specific marker for the NA2 allele of FcγRIIIb.

In most donors with at least one NA2 allele high mannose type species represented 80–90% of glycans at N45 from neutrophils but from only 40–60% in plasma derived FcγRIII based on relative abundance (Fig. 4*C*, 4*D*). This is not entirely unexpected as plasma derived FcγRIII is a mixture both FcγRIIIb and FcγRIIIa though most soluble FcγRIII has been reported to be FcγIIIb shed from neutrophils (29). The differences between neutrophil and plasma FcγRIII glycosylation at N45 held true for more than 90% of donors tested. However, two donors out of 50 had low levels (<10%) of high mannose glycans in isolates from neutrophils and two additional donors showed higher levels of high mannose glycans in plasma than on isolated neutrophils (Fig. 4*D*). The two donors with low levels of high mannose also had noncanonical FcγRIIIb alleles with the NA1/FcγRIIIa D65 variant replacing the N65 variant found in the NA2 allele suggesting an interaction between the glycans at N45 and the side chain of N65/D65 (Fig. 4*E*, 4*D*). The relative abundance of the individual glycopeptide varied substantially between donors (Table V).

**Fig. 5.**
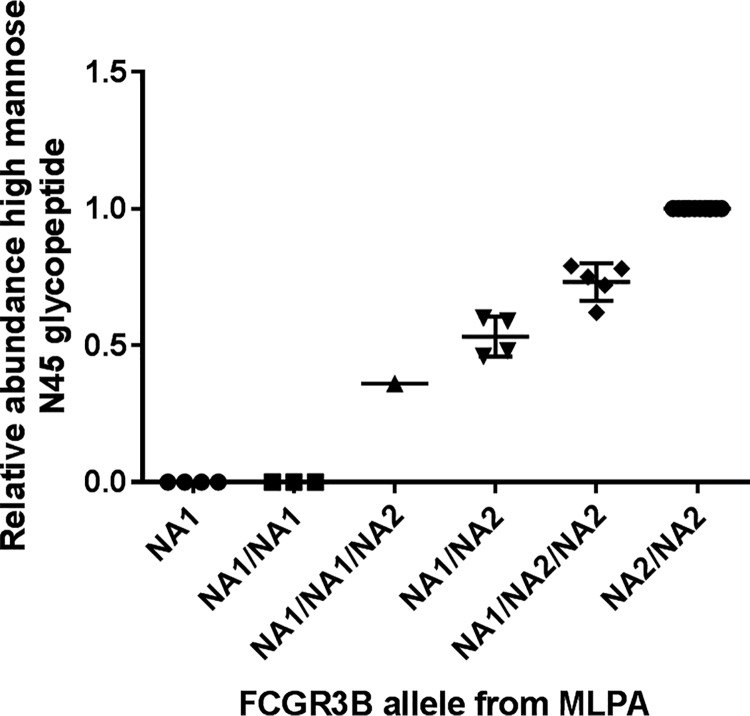
**High mannose glycans at N45 are specific to neutrophils and plasma abundance reflects allele distribution.** The relative abundance of high mannose type glycopeptides compared with the nonglycosylated NA1 peptide is significantly associated with the relative number of the two alleles (*p* < 0.0001 one way ordinary ANOVA).

The identification of FcγRIIIb specific high mannose type glycans allows for the determination of FcγRIIIb genotype and relative copy number of the two alleles where DNA/RNA is not available. Plotting the area abundance of high mannose type glycans in plasma relative to the nonglycosylated NA1 peptide for donors with different copy number variants shows a significant association between the levels of high mannose type glycans relative to the aglycosyl N45/N47 and the relative number of each allele as determined by MLPA (Fig. 5).

##### N162 Contains Complex Sialylated N-glycans With Variable Branching and Antennary Fucose

Glycopeptides at N162 were quantified from a combined chrymotrypsin endoproteinase GluC digest as described previously (19). Under these conditions only a single predominant peptide backbone with the sequence VGSKN(162)VSSE was formed (supplemental Fig. S1). Fourteen unique FcγRIIIb N162 glycopeptide masses were identified which eluted as 30 distinct chromatographic peaks. The N-glycans consist of complex type glycans which are predominantly sialylated and almost entirely core-fucosylated (Table VI). Sialylated N-glycans were found to contain both α2–6 and α2–3 linked sialic acid with the former being more abundant (Table VI). Antennary fucose was identified for a number of species including both Lewis and sialyl Lewis motifs based on the presence of characteristic nonreducing end fragments from the targeted MS/MS experiments (supplemental Fig. S4). Extraction of characteristic nonreducing end fragments from high resolution accurate mass (HR/AM) MS/MS allows for a clear differentiation between FA2G2F1S1 peak 1 and peak 3. From this analysis peak 1 is fucosylated on the nonsialylated antenna whereas peak 3 is fucosylated on the sialylated antenna. Fragmentation of the antennary fucosylated glycopeptide by low energy CID confirmed the assignments based on nonreducing fragments from HCD with HR/AM detection (supplemental Fig. S5). Interestingly there appears to be a preference for antennary sialylation of the α2–3 sialylated antennae consistent with a sLe^x^ structure (supplemental Fig. S8). These structures are likely to represent sLe^x^ not sLe^a^ structures based on the presence of the preferred sLe^x^ substrate Galβ1–4GlcNAc found on N-glycans (30).

**Table VI:**
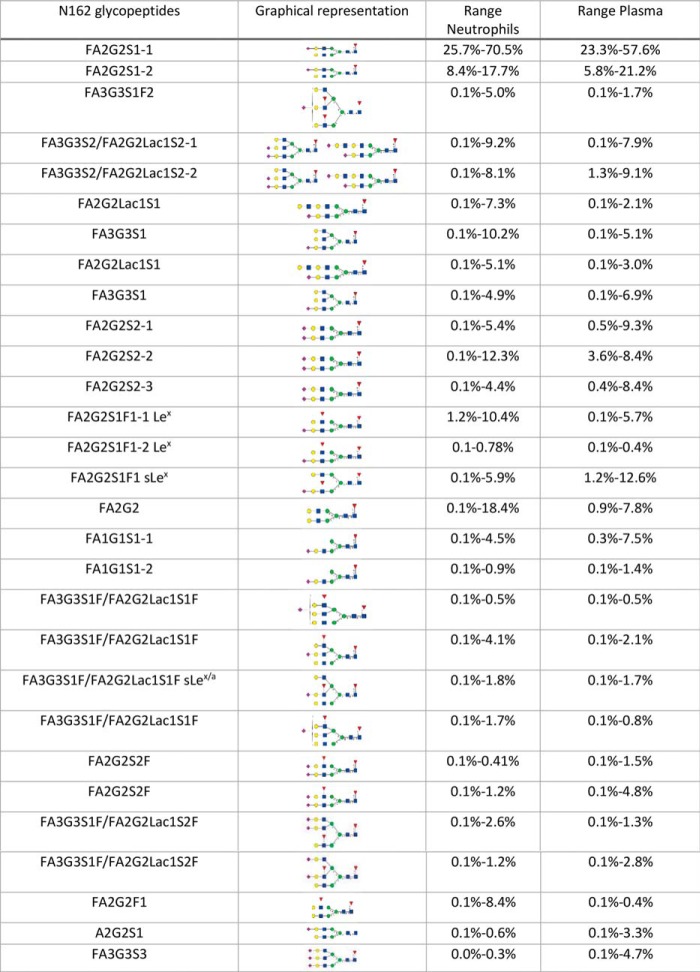
N162 N-glycosylation levels for neutrophil FcγRIIIb and plasma FcγRIIIa/b. Oxford nomenclature used for N162 glycopeptides column. Glycan structures were generated based on CFG guidelines using GlycoWorkBench. Multiple potential structures are shown when more detailed structural information could not be obtained

Highly branched structures and structures containing N-acetyllactosamine extensions were identified and characterized based on the detection of specific nonreducing end fragments. N-glycopeptides with up to three antennae and three sialic acids were identified as were species with N-acetyllactosamine extensions. One of the most abundant highly branched species was identified as FA3G3S1 (Table VI). Targeted MS/MS with extraction of diagnostic fragments from the nonreducing end reveals the presence of a fragment corresponding the di-lactosamine tetrasaccharide (diLacNAc) associated with the first chromatographic peak (supplemental Fig. S6). None of the 4 peaks generates a fragment suggesting the presence of sialylated di-LacNAc under HCD fragmentation. Low energy CID confirms the presence of di-LacNAc in the first peak (supplemental Fig. S7) as well as the absence of sialylated di-LacNAc even under lower energy fragmentation. There was substantial variation in the distribution of glycopeptide abundances at this site (Table VI). Similarly, to N45; N162 appears to be fully or highly occupied in these healthy donors.

##### The FcγRIIIb Allele Influences the Glycosylation at N162

**Fig. 6.**
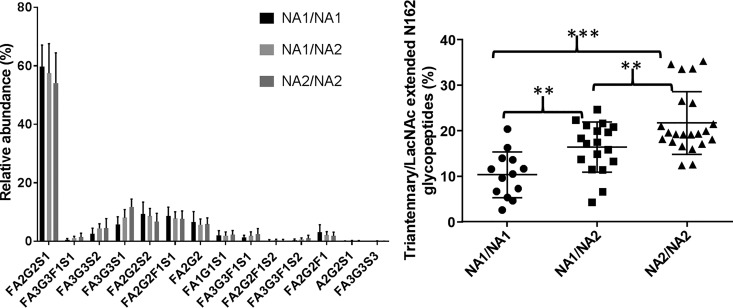
***A* Neutrophil FcγRIIIb N162 glycopeptide distribution comparison between FcγRIIIb alleles.** NA1/NA1 *n* = 14, NA1/NA2 *n* = 18, NA2/NA2 *n* = 19. *B*, Comparison of the relative abundance of Triantennary and lactosamine extended glycans between different FcγRIIIb alleles in healthy human donors. Turkey's multiple comparison test (** *p* < 0.01, *** *p* < 0.001 *t* test). The standard error is calculated for each allele based on a single replicate for each donor within the group.

Interestingly, a comparison between different FcγRIIIb polymorphic variants suggests glycosylation at N162 is influenced by the allele. Most of the glycans at N162 are biantennary however, a significant amount are triantennary or lactosamine extended species (Fig. 6*A*). A comparison of the N-glycopeptide relative abundances between alleles shows a trend toward increased levels of each of the branched species in the NA2 variant (Fig. 6*A*). When these similar glycoforms are combined there is a significant difference in the levels of these glycoforms across the different alleles (Fig. 6*B*). The association between the NA2 allele and higher levels of these larger glycopeptides is also seen when including copy number variants (supplemental Fig. S12). A comparison of the glycosylation patterns at N162 between paired neutrophil and plasma samples shows that plasma FcγRIII contains significantly lower levels of antennary fucose than the corresponding neutrophil sample (supplemental Fig. S14).

## DISCUSSION

We characterized FcγRIIIb glycosylation from isolated neutrophils as well as matched plasma, which contains both FcγRIIIb and FcγRIIIa, for donors with different FcγRIIIb genotypes. The N45 glycosylation site of FcγRIIIb from neutrophils was found to contain primarily high mannose type glycans with lower levels of sialylated hybrid structures. This site in FcγRIIIb is specifically found on the NA2 allele and high mannose species were absent from soluble FcγRIII from donors homozygous for the NA1 allele suggesting the presence of these species in plasma is a marker for the NA2 allele. The allele specific nature of the high mannose type glycans we observed is consistent with previous reports which showed Concanavalin-A (Con-A) binding specifically to the NA2 allele of FcγRIIIb (31), (32). The NA2 specific glycosylation site N45 is likely responsible for the allele specific Con-A reactivity observed by these authors based on the lack of occupancy we observed at N65 the other NA2 specific site.

Previous work utilizing lectin binding identified cell type specific glycosylation of FcγRIIIa. In this study FcγRIIIa from NK cells was found to contain both high mannose and complex type glycans whereas monocyte FcγRIIIa was found to contain only complex type glycans (32). The authors utilized Con-A reactivity to characterize the glycosylation and many of the hybrid type structures found on soluble FcγRIIIa from donors homozygous for the NA1 allele would also bind the Con-A lectin (33). It may be that these hybrid structures are responsible for Con-A reactivity seen in NK cell derived FcγRIIIa. A recent report analyzing glycans released from FcγRIIIa isolated from NK cells identified high mannose type glycans though the specific site was not determined (34). This raises the possibility that FcγRIIIa from NK cells contains high mannose glycans at a site that was not analyzed in this study. It is also possible that in healthy donors very little sFcγRIII comes from NK cells which is consistent with their low frequency in peripheral blood.

The abundance of these high mannose type N45 glycopeptides in plasma relative to the NA1 specific aglycosyl peptide is proportional to the relative number of the two alleles as measured by MLPA which supports the finding that high mannose type glycans at N45 on soluble FcγRIII are a marker for the NA2 allele. This method of assigning genotype showed high concordance with MLPA using DNA from isolated neutrophils. The high concordance coupled with the low volume of plasma required for analysis suggest that this approach could be applied to characterize FcγRIIIb alleles from plasma in translational studies. In fact, we have utilized this approach to assign FcγRIIIb alleles in plasma samples from RA patients treated with anti-TNFα therapy. This has allowed us to evaluate the relationship between FcγRIIIb alleles and response to anti-TNFα therapy.

We observed an association between the amino acid at position 65 and the glycosylation at N45. The combination of N45/D65 was associated with predominantly sialylated hybrid structures at N45 whereas the canonical NA2 N45/N65 combination resulted in predominantly high mannose type glycans at N45. Interestingly, FcγRIIIa which has the N45/D65 combination contains sialylated hybrid type structures but not high mannose type structures at N45. The N65 residue was found to be largely unoccupied. Examining the crystal structure of glycosylated FcγRIIIa interactions between the glycans at N45 and the side chain of D65 are apparent (14). Solution NMR analysis of FcγRIIIa confirms that the glycans at N45 interact strongly with the protein backbone around N65 (16). This glycan protein interaction inhibits glycosylation of this site resulting in the observed lack of site occupancy of N65.

Comparing the glycosylation of FcγRIIIb from neutrophil and FcγRIII from matched plasma revealed differences between the cell surface and soluble glycoforms at both sites. Lower levels of high mannose type glycosylation at N45 and lower levels of antennary fucose at N162 were seen in plasma compared with isolated neutrophils. It is somewhat surprising that the glycosylation patterns between neutrophil FcγRIIIb and soluble FcγRIII are notably different given that most soluble FcγRIII has been reported to be neutrophil derived (29). The higher abundance of the sialylated species at N45 in plasma relative to neutrophils could result from preferential clearance of high mannose bearing FcγRIII. High mannose type glycans have been shown to interact with the mannose receptor resulting in reduced serum half-life for high mannose bearing glycoproteins (35). The lower levels of antennary fucose observed in plasma compared with matched isolated neutrophils could arise from similar phenomenon. Neutrophil granule proteins bearing Lewis^X^ structures were cleared from serum by scavenger receptor C-type lectin (SCLR) (36). Site specific characterization of FcγRIIIa from isolated cell populations may help detangle the site specific glycosylation pattern for different cell types.

We have identified an association between the glycosylation pattern at this site and the FcγRIIIb allele. Comparing the levels of highly branched glycans at this sites reveals significantly higher levels of these bulky glycans are present in the NA2 allele. This effect is somewhat surprising as variant residues for the allele are found in the first Ig like domain while this glycosylation site is found in the second Ig domain. However, recent reports noting a distant interaction between the glycans at N45 and the residues in and around N162 may help explain this association (16). The N-glycans at N162 are found at the Fc:FcγR binding interface and it has been proposed that differences in glycosylation at this site would have the most significant effect on the interaction (14), (19), (20).

Previous comparisons of the interaction kinetics between IgG and FcγRIIIa produced in different cell lines suggests more highly branched species as seen in the NA2 allele may have a destabilizing effect on the Fc:FcγR interaction. A faster dissociation rate for IgG binding to recombinant FcγRIIIa expressed in different cell lines was associated with higher levels of highly branched and sialylated glycans (20). In examining the influence of FcγR glycosylation on IgG binding we observed that enzymatic desialylation of recombinant FcγRIIIa expressed in CHO cells (supplemental Fig. S14, S15) resulted in a 2-fold increase in equilibrium affinity (supplemental Fig. S16) suggesting glycosylation can also influence affinity.

The allelic influence on the glycosylation profile is intriguing in light of functional studies examining the influence of FcγRIIIb allele on FcγR mediated neutrophil activation. These studies have reported a greater response to several stimuli has been reported for neutrophils bearing the NA1 allele (7), (8), and (9). Unlike the well-studied polymorphic variants of FcγRIIIa and FcγRIIa no differences in affinity have been seen between IgG and the recombinant FcγRIIIb variants (3). The expression system used to produce recombinant FcγRs has a strong effect of the glycosylation profile but it is unclear if the allelic differences in glycosylation we observed in endogenous FcγRIIIb are reflected in the allelic variants of the recombinant protein. Our results coupled with the demonstrated influence of recombinant FcγRIIIa glycosylation on the kinetics of the Fc:FcγRIIIa interaction raise the possibility that the differences in neutrophil activity for these two alleles may be influenced by differences in glycosylation.

## DATA AVAILABILITY

The raw data can be found at http://www.peptideatlas.org/PASS/PASS01275, Deposit ID: PASS01275.

## Supplementary Material

Supplemental Data

## Footnotes

^1^ The abbreviations used are:

FcγR: receptor for Fc region of IgG
ADCP: antibody dependent cellular phagocytosis
ADCC: antibody dependent cellular cytotoxicity
ITAM: immunoreceptor tyrosine-based activation motif
GPI: glycophosphatidylinositol
MLPA: multiplexed ligation-dependent probe amplification.
