# Supplementary material for: Characterization of Endogenous Human FcγRIII by Mass Spectrometry Reveals Site, Allele and Sequence Specific Glycosylation*
Source: Mol Cell Proteomics. 2018 Dec 17;18(3):534–45. doi: 10.1074/mcp.RA118.001142 (PMC6398215; doi:10.1074/mcp.RA118.001142)
Supplement: Supplemental Data [file 141355_1_supp_238789_pdk5kh.pdf]

Analysis of recombinant FcγRIIIa glycosylation from different cell types

The glycosylation pattern of rFcγRIII N45, was characterized using a chymotryptic digestion (Sequencing Grade Promega V1061) to generate the glycopeptide shown in table 1. The glycosylation at N162 was analyzed following sequential digestion with endoproteinase GluC (Sequencing Grade Promega V1651) followed by chymotrypsin. The peptides and glycopeptides were analyzed by nLC-MS/MS on a Dionex Ultimate 3000 nano RSLC (ThermoFisher Scientific) coupled to a QExactive mass spectrometer (ThermoFisher Scientific) equipped with and EasySpray nano-LC source (ThermoFisher Scientific). Peptides were separated on an EasySpray C18 column (0.75x250mm 2 µm particle size ThermoFisher Scientific ES802). A data dependent acquisition was run to identify glycopeptides from each site. Glycopeptides were identified by searching the high resolution accurate mass MS/MS spectra for Y-1 ions which tend to form readily under HCD fragmentation. Glycopeptides were quantified based on the extracted ion area of the most abundant charge state for each identified species.

Surface Plasmon Resonance (SPR)

Equilibrium binding assays were carried out on a ProteOn XPR36 system. Briefly, Fc probes were immobilized to a GLC sensor surface by direct amine coupling and passed over with receptors in PBS-T0.05% running buffer at 30µL/min. At least three replicates were measured per receptor. The probe surface was regenerated with an 18s pulse of 10mM glycine, pH 1.5 in between analyte injections. Data was analyzed in ProteOn Manager software.

Desialylation of highly sialylated CHO produced FcγRIIIa results in increased affinity for IgG

It is well established that protein glycosylation is impacted by the choice of expression system. This has been shown for multiple proteins [34] and specifically for FcγRIIIa [19], [20]. The different glycosylation patterns of FcγRIIIa produced in different expression systems was determined to influence the kinetics of the Fc:FcγRIIIa interaction. We have characterized glycosylation of FcγRIIIa from HEK and CHO cells specifically focusing on the glycosylation at N45 and N162 two sites. We characterized the N162 site which is found at the binding interface between IgG Fc and FcγRIII. We also characterized the N45 site which was demonstrated to influence the interaction [12] though apparently not through direct contact with the Fc.

The glycosylation of both recombinant proteins was largely similar to what has been previously reported [19], [20]. Differences between the expression systems were found to be specific to each of the two sites. The N-glycans at FcγRIII N162 of the CHO material were primarily biantennary complex type glycans terminating in sialic acids (Figure S15). The N162 glycans of the HEK produced protein were predominantly complex type biantennary structures terminating in GalNAc with high levels of antennary fucose and low levels of sialic acid (Figure S15). Desialylation of the CHO-produced protein generated a protein with ~90% G2F at this site. The glycosylation at N45 was also distinct for the two expression systems. The CHO-produced material contained predominantly binatennary complex type glycans terminating in sialic acid with lower levels of hybrid type glycans (Figure S15). Compared to N162 the glycans at N45 contained more branching, less fucose and contained hybrid type glycans which were absent from N162. In contrast to N162 only low levels of non-reducing end GalNAc were seen in the HEK

material with most glycans terminating in sialic acid (Figure S14). Desialylation of the CHO material resulted in a protein containing ~35% G2F as the predominant species at N45 (Figure S14).

A direct comparison between the recombinantly expressed FcγRIIIa and neutrophil FcγRIIIb shows the most substantial differences in glycosylation at N45. At this site complex species predominate in the recombinant protein while high mannose and hybrid type species are the predominant species at this site from FcγRIIIb isolated from neutrophils (Figure S17). The differences seen here may be due to the different protein source but also to the fact that these represent distinct isoforms of CD16 (FcγRIII). The glycosylation at N162 is fairly similar between native FcγRIIIb isolated from neutrophils and FcγRIIIa expressed in CHO cells. Differences are seen in the degree of branching which was lower in the CHO expressed protein (Figure S18).

Previous work demonstrated the influence of FcγRIII glycosylation on the affinity and the kinetics of the FcγR-IgG interaction [19], [20]. Both groups had reported similar affinity for proteins from the two expression systems but with significantly different kinetics. We set out to determine the effect of removal of sialic acid on the equilibrium affinity of FcγR-IgG interaction. Based on the equilibrium binding data it appears that removal of the sialic acids had a substantial influence on the affinity particularly when put into the context of the well-studied V158F polymorphism. The V158 variant expressed in HEK cells has about 1.5x higher affinity for monomeric Fc compared to the F158 variant expressed in HEK which is consistent with previous reports [3]. As reported previously the affinity constant is not substantially influenced by the expression system. However, removal of sialic acid from the V158 material produced in CHO cells increased the affinity dramatically (Figure S17). The effect of sialic acid removal on affinity was larger than that observed for the increased affinity of polymorphism V158 relative F158.These results are intriguing and further support the role of FcγRIIIa glycosylation in modulating the FcγR-IgG interaction.

Supplementary Materials:

Supplemental Table

Table S1: Comparison of FCGR3B alleles assigned by mass spectrometry and MLPA. The NA2 allele was identified based on the presence of high mannose type glycans at N45 while the NA1 allele was identified based on the presence of the non-glycosylated peptide. Four donors were identified with non-canonical residues. Two donors (*) homozygous for the NA1 allele at N45/N47 were found to have the NA2 I89 variant. Two additional donors (#) homozygous for the NA2 allele at N45/S47 were found to have the NA1 variant D65. The presence of the NA1 variant D65 in these donors was associated with higher levels of sialylated N-glycans and lower levels of high mannose type glycans at N45.

Donor	FcγRIIIb allele Glycoprotein	FcγRIIIb Allele and CNV MLPA	Copies FCGR3B
**Donor-1**	NA1/NA1		
**Donor-2**	NA1/NA1	NA1	1
**Donor-3**	NA1/NA1	NA1	1
**Donor-4**	NA1/NA1		
**Donor-5**	NA1/NA1		
**Donor-6**	NA1/NA1		
**Donor-7**	NA1/NA1		
**Donor-8**	NA1/NA1	NA1	1
**Donor-9**	NA1/NA1		
**Donor-10**	NA1/NA1	NA1	1
**Donor-11**	NA1/NA1	NA1/NA1	2
**Donor-12**	NA1/NA1	NA1/NA1	2
**Donor-13**	NA1/NA1*	NA2/NA2	2
**Donor-14**	NA1/NA1*		
**Donor-15**	NA1/NA2		
**Donor-16**	NA1/NA2		
**Donor-17**	NA1/NA2		
**Donor-18**	NA1/NA2		
**Donor-19**	NA1/NA2		
**Donor-20**	NA1/NA2		
**Donor-21**	NA1/NA2	NA1/NA2	2
**Donor-22**	NA1/NA2	NA1/NA2	2
**Donor-23**	NA1/NA2	NA1/NA2	2

**Donor-24**	NA1/NA2		
**Donor-25**	NA1/NA2	NA1/NA2	2
**Donor-26**	NA1/NA2		
**Donor-27**	NA1/NA2	NA1/NA2/NA2	3
**Donor-28**	NA1/NA2	NA1/NA2/NA2	3
**Donor-29**	NA1/NA2	NA1/NA2/NA2	3
**Donor-30**	NA1/NA2	NA1/NA1/NA2	3
**Donor-31**	NA1/NA2		
**Donor-32**	NA1/SH		
**Donor-33**	NA1/SH		
**Donor-34**	NA2/NA2		
**Donor-35**	NA2/NA2	NA2/NA2	2
**Donor-36**	NA2/NA2		
**Donor-37**	NA2/NA2#		
**Donor-38**	NA2/NA2	NA2/NA2	2
**Donor-39**	NA2/NA2	NA2/NA2	2
**Donor-40**	NA2/NA2	NA2/NA2	2
**Donor-41**	NA2/NA2		
**Donor-42**	NA2/NA2	NA2/NA2	2
**Donor-43**	NA2/NA2	NA2/NA2	2
**Donor-44**	NA2/NA2		
**Donor-45**	NA2/NA2#		
**Donor-46**	NA2/NA2		
**Donor-47**	NA2/NA2		

**Donor-48**	NA2/NA2	NA2/NA2	2
**Donor 49**	NA2/NA2		
**Donor 50**	NA2/NA2		

Table S2: Precursor sequence and fragment ions monitored for the quantitation of peptides and glycopeptides by targeted MS/MS

protein_name	transition_name	sequence	prec_z	frg_type	frg_nr	frg_z	Fragment m/z
**FcγRIIIb NA1/FcγRIIIa**	FcγRIII A61/D65	FIDAATVDDSGEY	2	y	6	1	685.23
**FcγRIIIb NA2**	FcγRIII A61/N65	FIDAATVNDSGEY	2	y	6	1	684.25
**FcγRIIIb SH**	FcγRIII D61/N65	FIDDATVNDSGEY	2	y	6	1	684.25
**FcγRIIIb NA1**	FcγRIII V89	SDPVQLEVHVGW	2	y	10	2	582.32
**FcγRIIIb NA2/SH/FcγRIIIa**	FcγRIII I89	SDPVQLEVHIGW	2	y	10	2	589.32
**FcγRIIIb NA1**	FcγRIII N47	FHNENLISSQASSY	2	b	7	1	868.43
**FcγRIIIb NA2**	FcγRIIIb NA2 M5	FHN(1216.42)ESLI SSQASSY	2	Y	1	1	1772.8
**FcγRIIIb NA2**	FcγRIIIb NA2 M6	FHN(1378.47)ESLI SSQASSY	2	Y	1	1	1772.8
**FcγRIIIb NA2**	FcγRIIIb NA2 M7	FHN(1540.53ESLIS SQASSY	2	Y	1	1	1772.8
**FcγRIIIb NA2**	FcγRIIIb NA2 M8	FHN(1702.58)ESLI SSQASSY	2	Y	1	1	1772.8
**FcγRIIIb NA2**	FcγRIIIb NA2 M9	FHN(1864.64)ESLI SSQASSY	2	Y	1	1	1772.8
**FcγRIIIb NA2/FcγRIIIa**	FcγRIII S47 N45 M4A1G1S1	FHN(1710.57)ESLI SSQASSY	3	Y	1	1	1772.8
**FcγRIIIb NA2/FcγRIIIa**	FcγRIII S47 N45 FM4A1G1S1	FHN(1856.66)ESLI SSQASSY	3	Y	1	1	1772.8
**FcγRIIIb NA2/FcγRIIIa**	FcγRIII S47 N45 M5A1G1S1	FHN(2018.71)ESLI SSQASSY	3	Y	1	1	1772.8
**FcγRIIIb NA2/FcγRIIIa**	FcγRIII S47 N45 A1G1S1	FHN(1548.54)ESLI SSQASSY	3	Y	1	1	1772.8
**FcγRIIIb NA2/FcγRIIIa**	FcγRIII S47 N45 FA1G1S1	FHN(1694.60)ESLI SSQASSY	3	Y	1	1	1772.8
**FcγRIII**	FcγRIII N162 FA2G2S1F1	VGSKN(2205.79)V SSE	3	Y	1	1	1109.53

**FcγRIII**	FcγRIII N162 FA3G3S1	VGSKN(2424.87)VSSE	3	Y	1	1	1109.53
**FcγRIII**	FcγRIII N162 FA3G3S1F1	VGSKN(2862.02)VSSE	3	Y	1	1	1109.53
**FcγRIII**	FcγRIII N162 FA3G3S2	VGSKN(2715.96)VSSE	3	Y	1	1	1109.53
**FcγRIII**	FcγRIII N162 FA1G1S1	VGSKN(1694.60)VSSE	3	Y	1	1	1109.53
**FcγRIII**	FcγRIII N162 FA2G2S1	VGSKN(2069.74)VSSE	3	Y	1	1	1109.53
**FcγRIII**	FcγRIII N162 FA2G2	VGSKN(1768.64)VSSE	3	Y	1	1	1109.53
**FcγRIII**	FcγRIII N162 FA2G2S2	VGSKN(2350.83)VSSE	3	Y	1	1	1109.53
**FcγRIII**	FcγRIII N162 FA2G2S2F1	VGSKN(2496.89)VSSE	3	Y	1	1	1109.53
**FcγRIII**	FcγRIII N162 FA3G3S2F1	VGSKN(2862.02)VSSE	3	Y	1	1	1109.53
**FcγRIII**	FcγRIII N162 FA2G2F1	VGSKN(1914.70)VSSE	3	Y	1	1	1109.53
**FcγRIII**	FcγRIII N162 FA3G3S3	VGSKN(3007.06)VSSE	3	Y	1	1	1109.53
**FcγRIII**	FcγRIII N162 Agycosyl	VGSKNVSSE	3	b	5	1	486.27

Supplemental Figures:

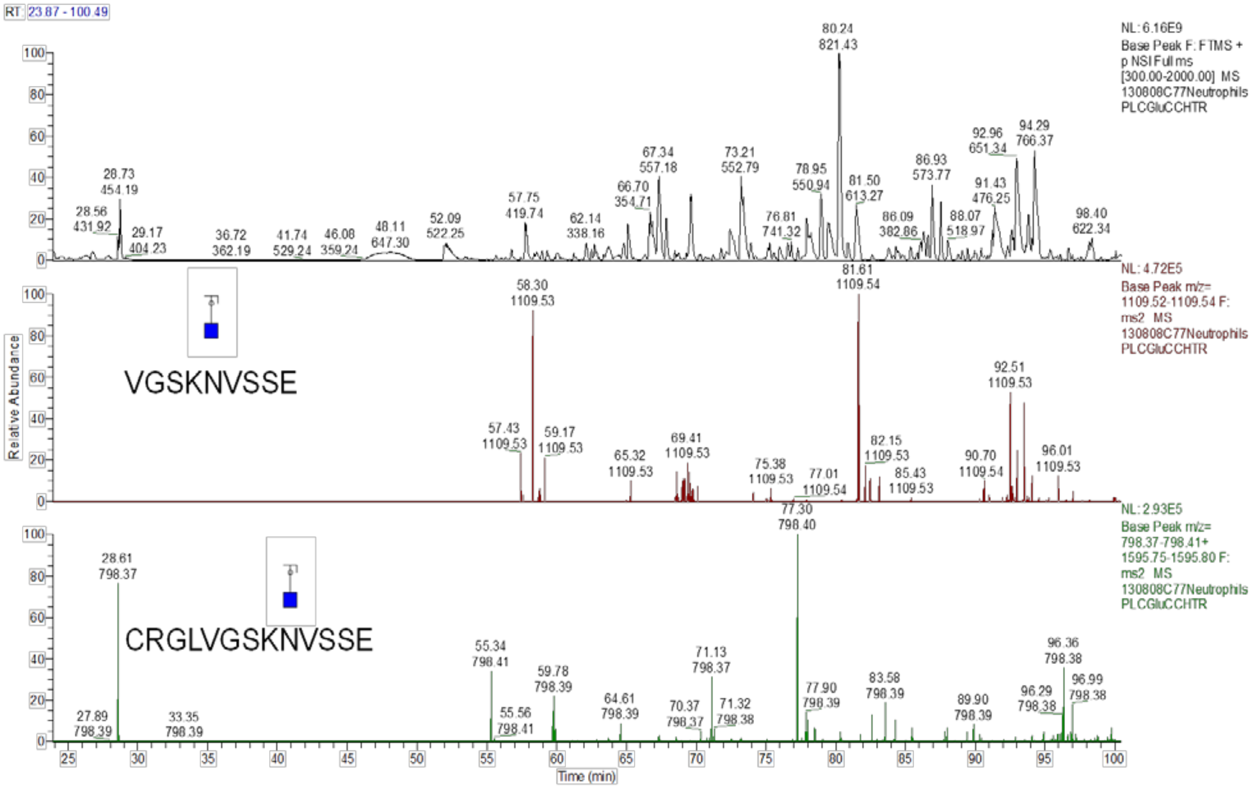

Figure S1. Y1 fragment ion scanning to identify N162 glycopeptides. Only a single proteolytic species was identified with the sequence VGSKNVSSE

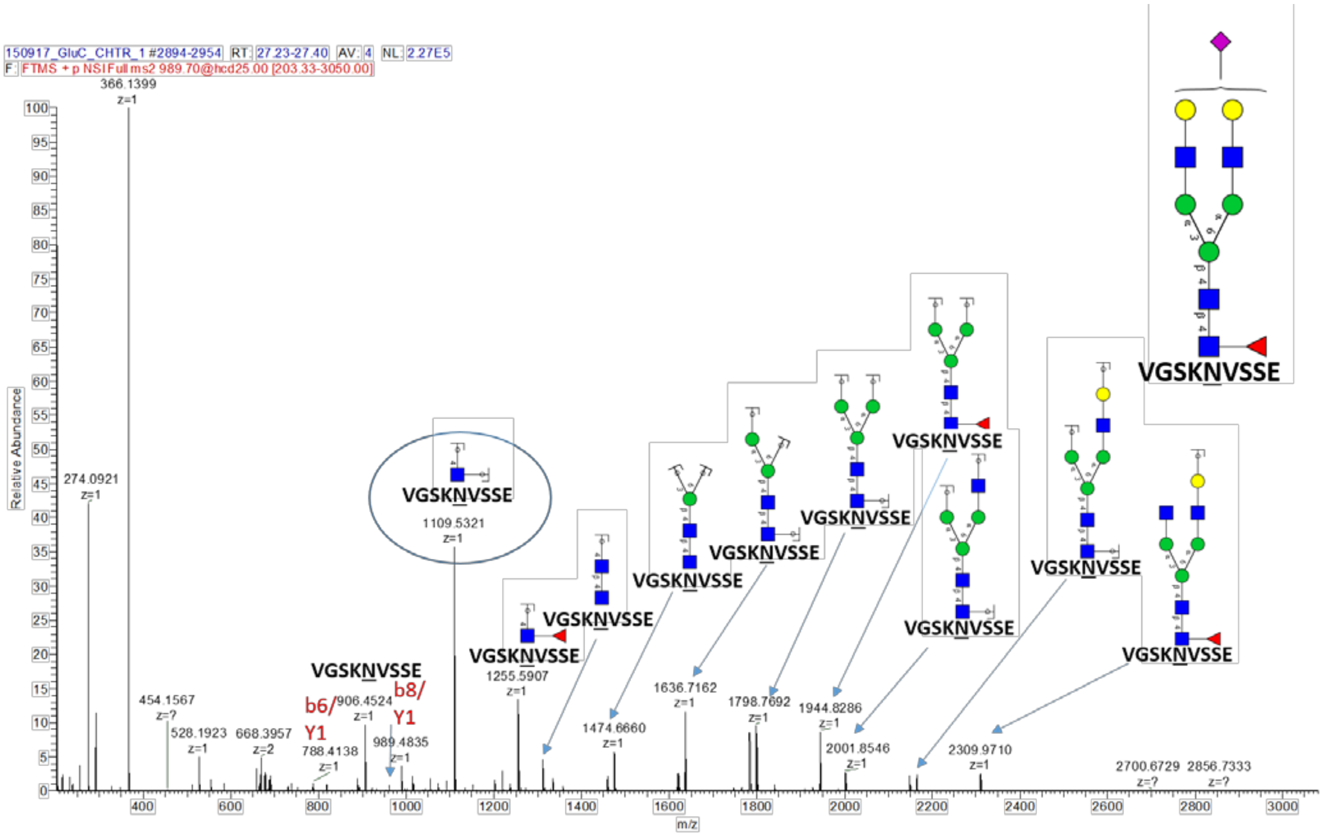

Figure S2. MS/MS spectrum of FcγRIIIb N162 FA2G2S1 the Y1 marker ion used to quantify N162 glycopeptides is circled.

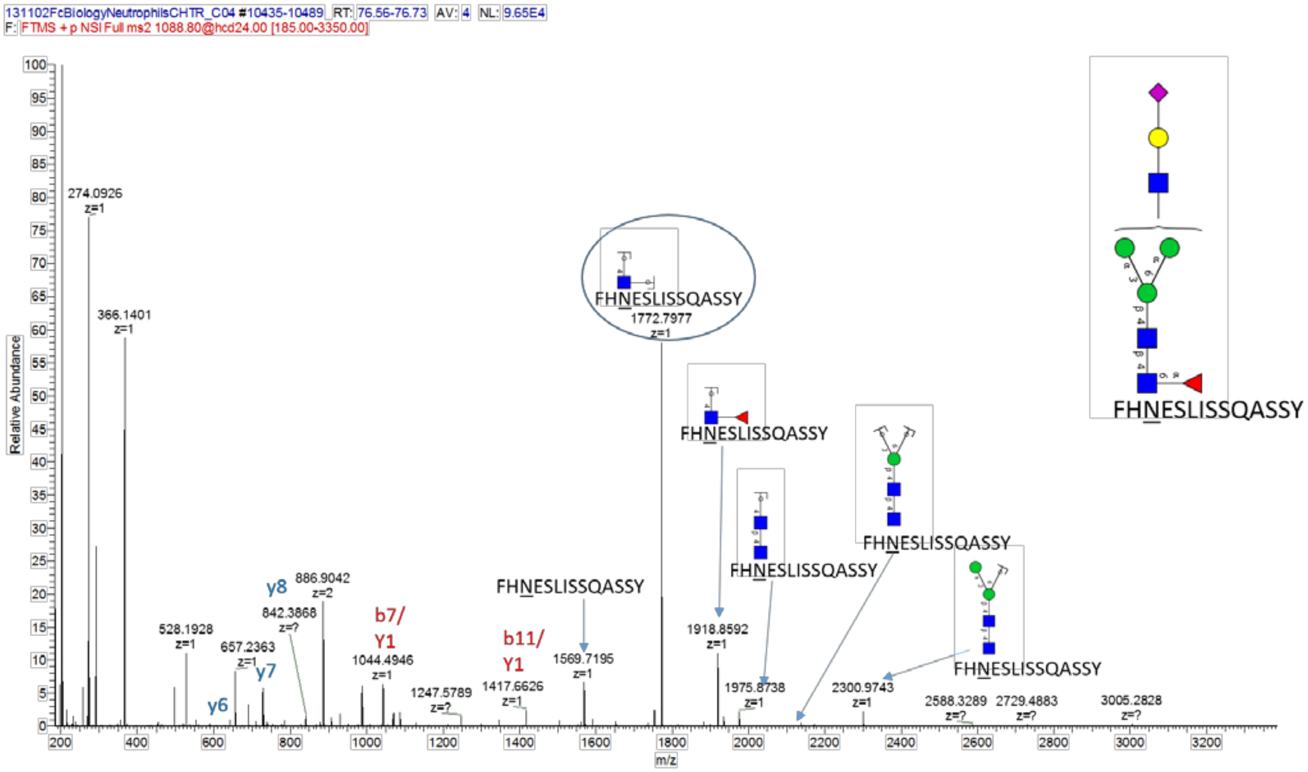

Figure S3. MS/MS spectrum of FcγRIIIb N45 M7 the Y1 marker ion used to quantify N45 glycopeptides is circled.

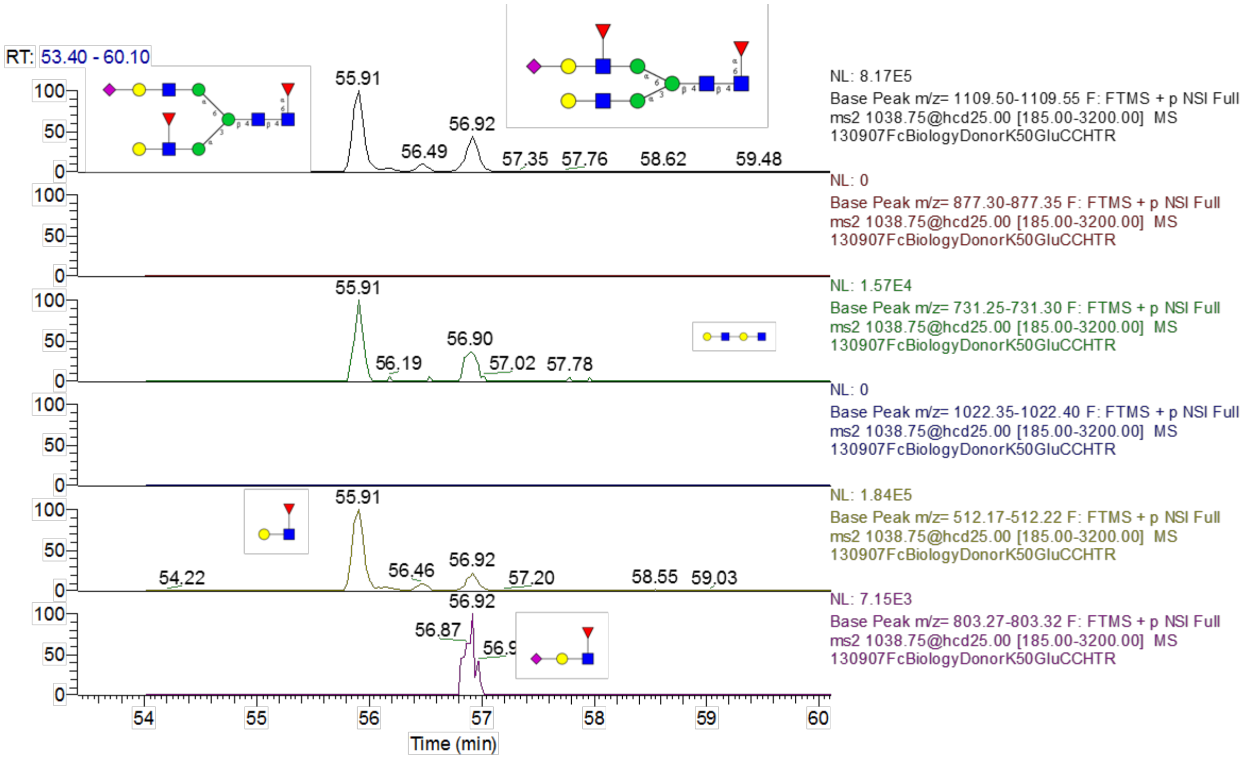

Figure S4. Non-reducing end fragment extracted ion chromatogram from HCD MS/MS of FA2G2F1S1. Peak 1 and peak 2 correspond to a structure having the fucose on the non-sialylated branch (Le^x) while peak 3 corresponds to a structure having fucose on the sialylated branch (sLe^x).

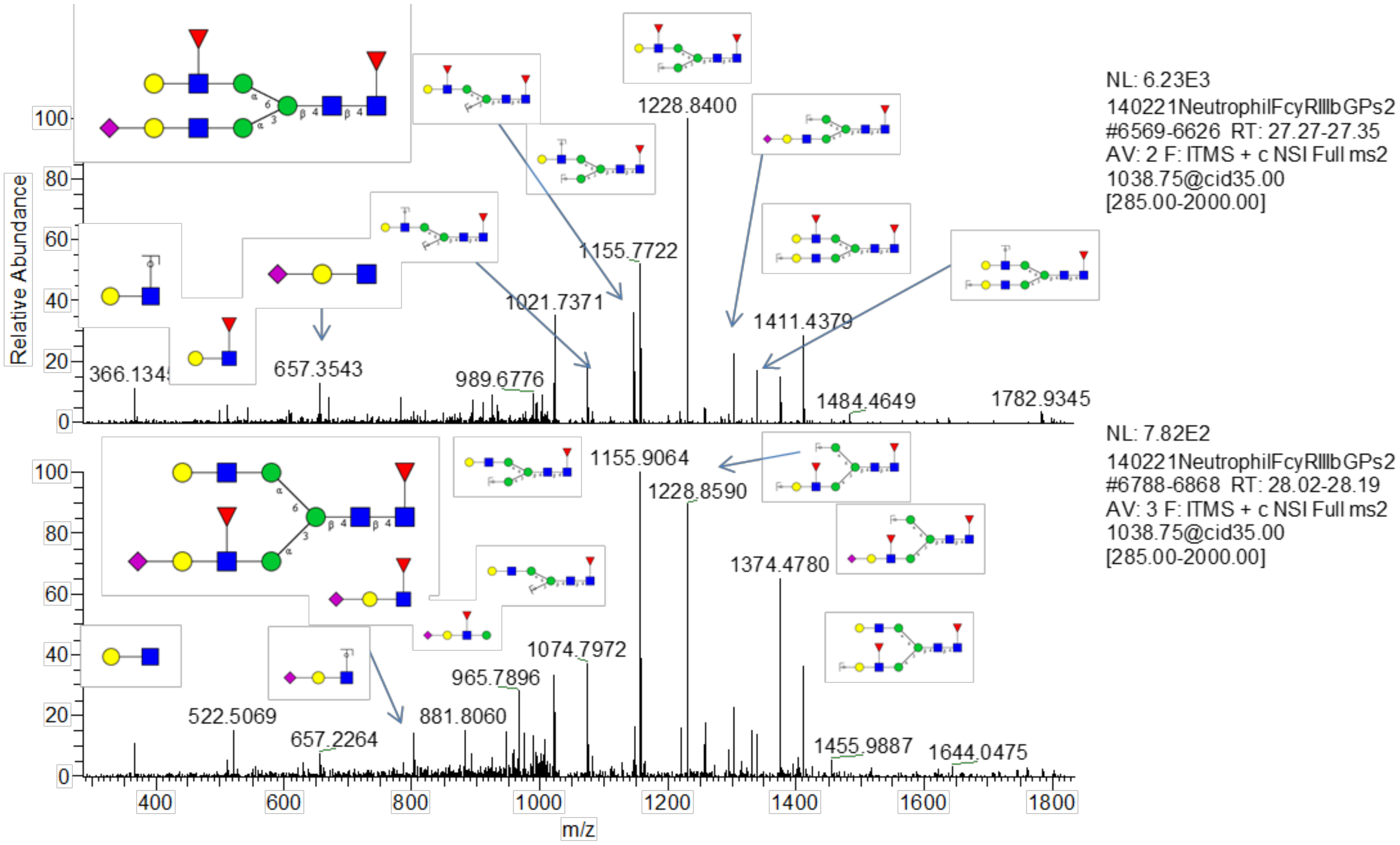

Figure S5. CID MS/MS fragmentation of FA2G2F1S1Peak 1 and peak 3. The CID fragmentation corroborates the structures suggested by the non-reducing end fragments. Peak 1 correspond to a structure having the fucose on the non-sialylated branch (Le^x) while peak 3 corresponds to a structure having fucose on the sialylated branch (sLe^x).

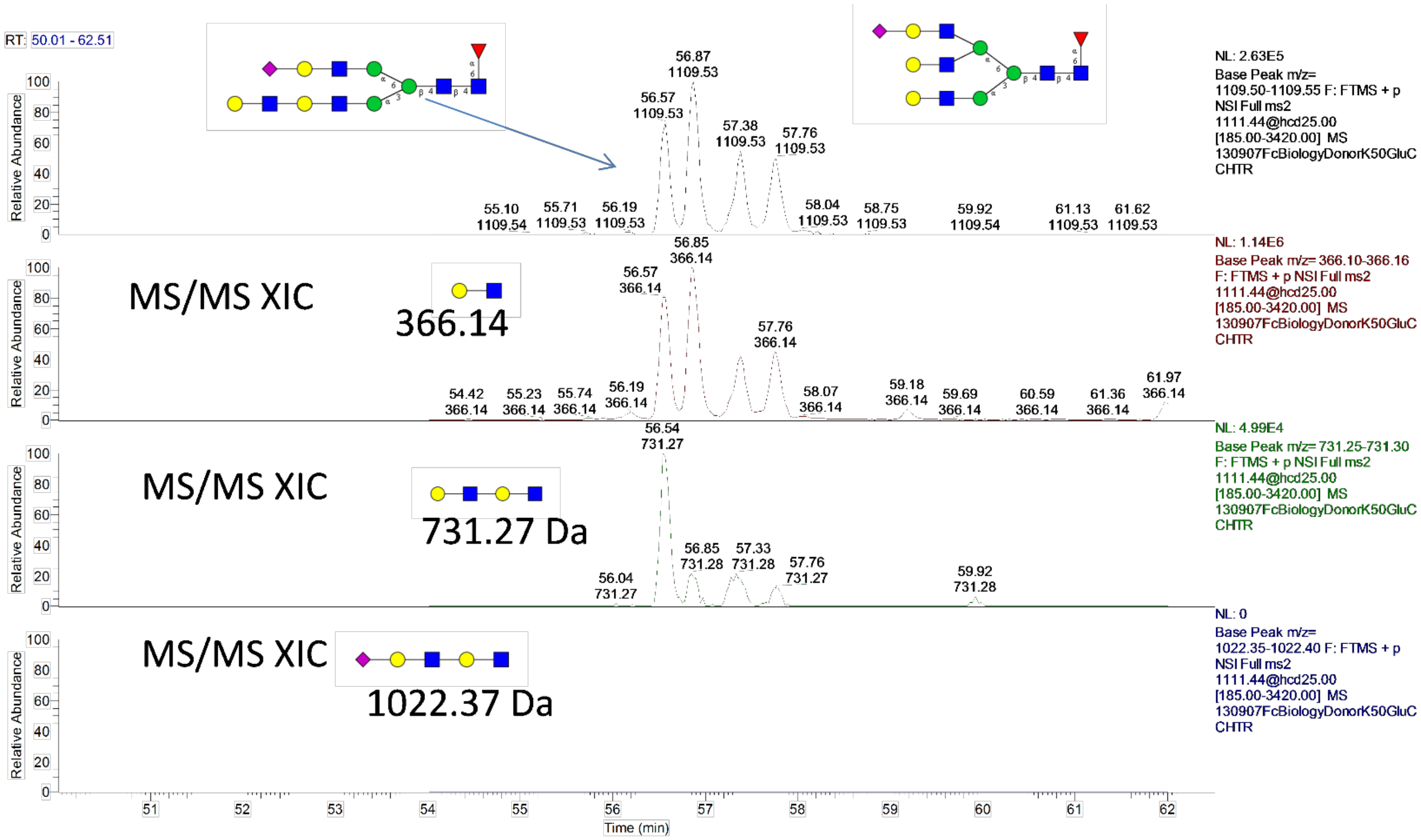

Figure S6. Non-reducing end fragment extracted ion chromatogram from HCD MS/MS of FA3G3S1. The MS/MS B-ion extracted ion chromatogram suggests peak 1 contains an N-acetyllactosamine extension based on the abundance of the fragment with m/z 731.27 at this retention time. The sialylated N-acetyllactosamine structure is not observed at all; this species may not be sufficiently stable under HCD fragmentation.

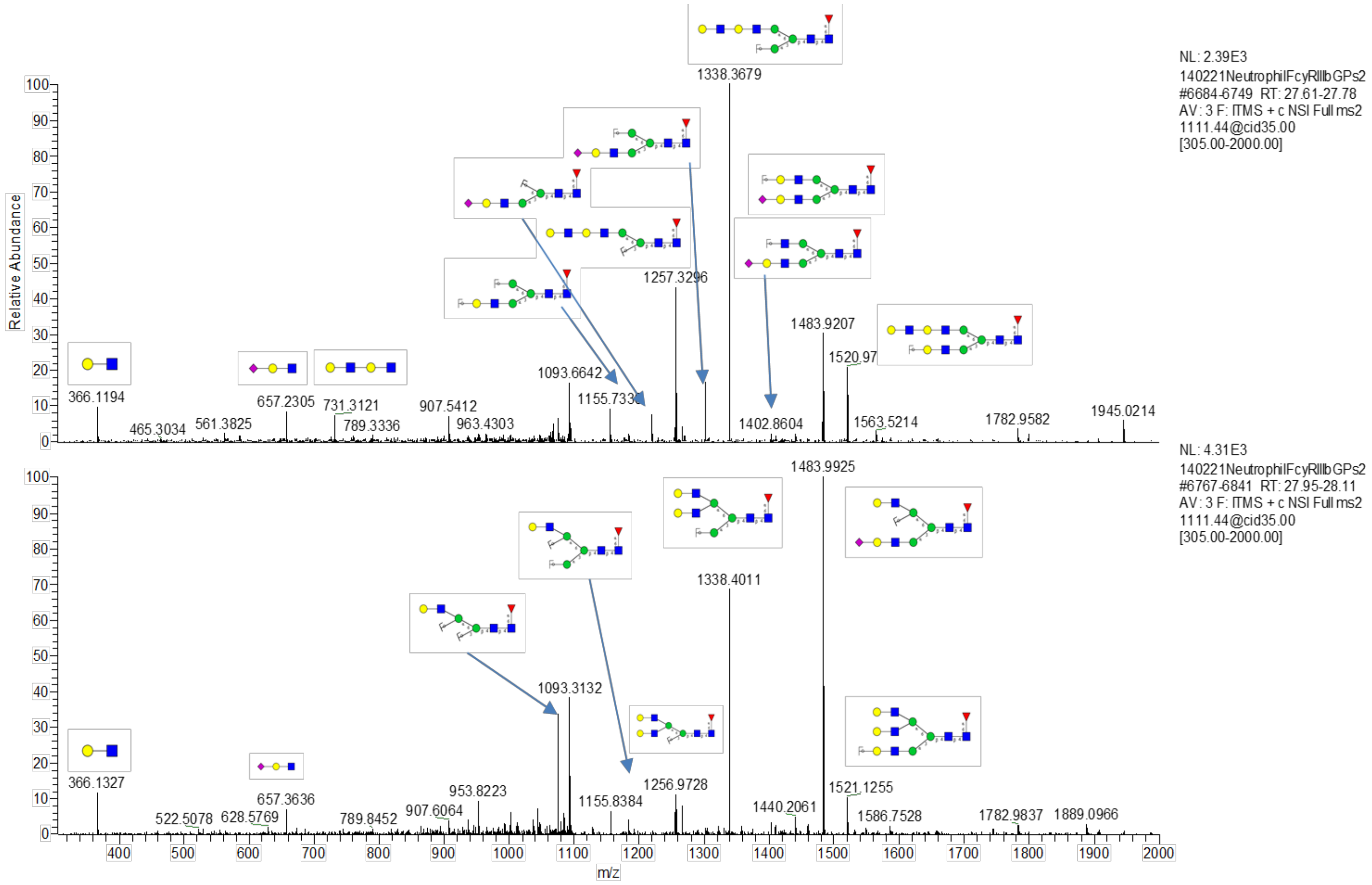

Figure S7. CID MS/MS of peak 1 and peak 2 corresponding to FA3G3S1. The low energy CID MS/MS spectrum confirms that peak 1 contains N-acetyllactosamine. The sialylated N-acetyllactosamine fragment was not detected in the CID MS/MS spectrum either.

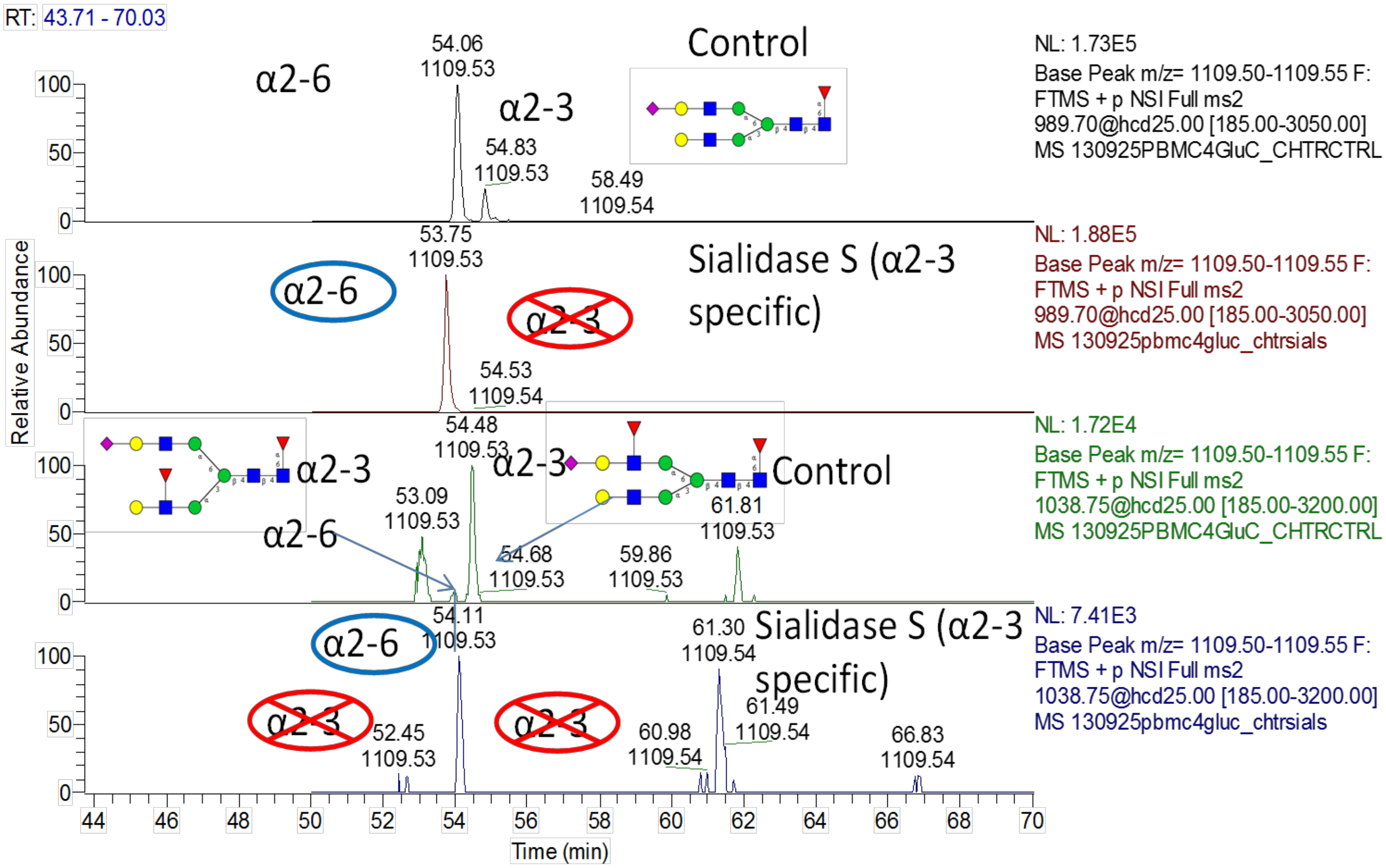

Figure S8. Sialidase treatment of N162 glycopeptides reveals both α2-3 and α2-6 linked sialic acid. The α2-3 linked sialic acid results in a slight shift to later retention time compared to the α2-6 linked sialic acid.

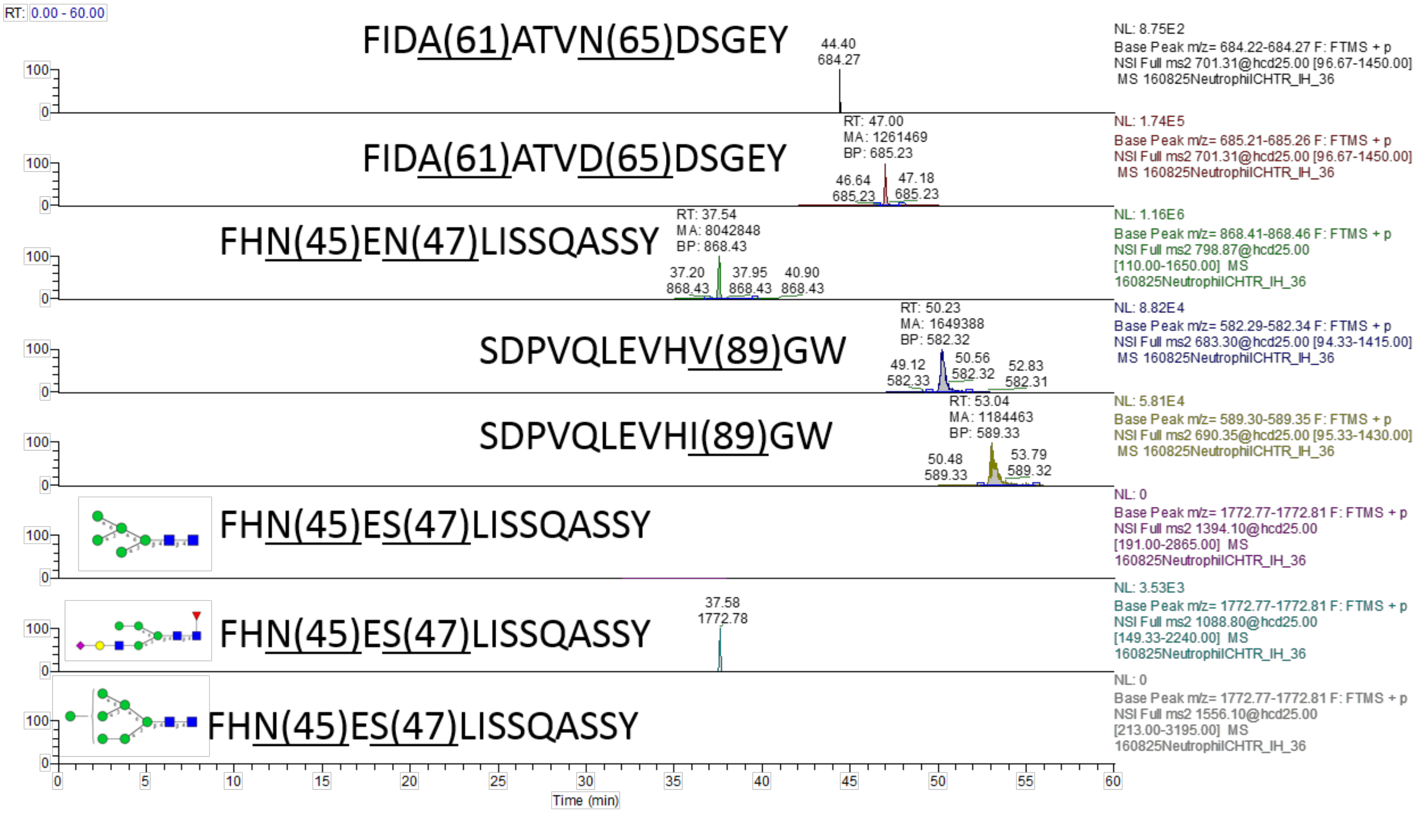

Figure S9*:* Example of maker peptides identified from a non-canonical NA1/NA1 donor. This donor has only the NA1 specific non-glycosylated N45/N47 at this site but also has the NA2 specific I89 peptide.

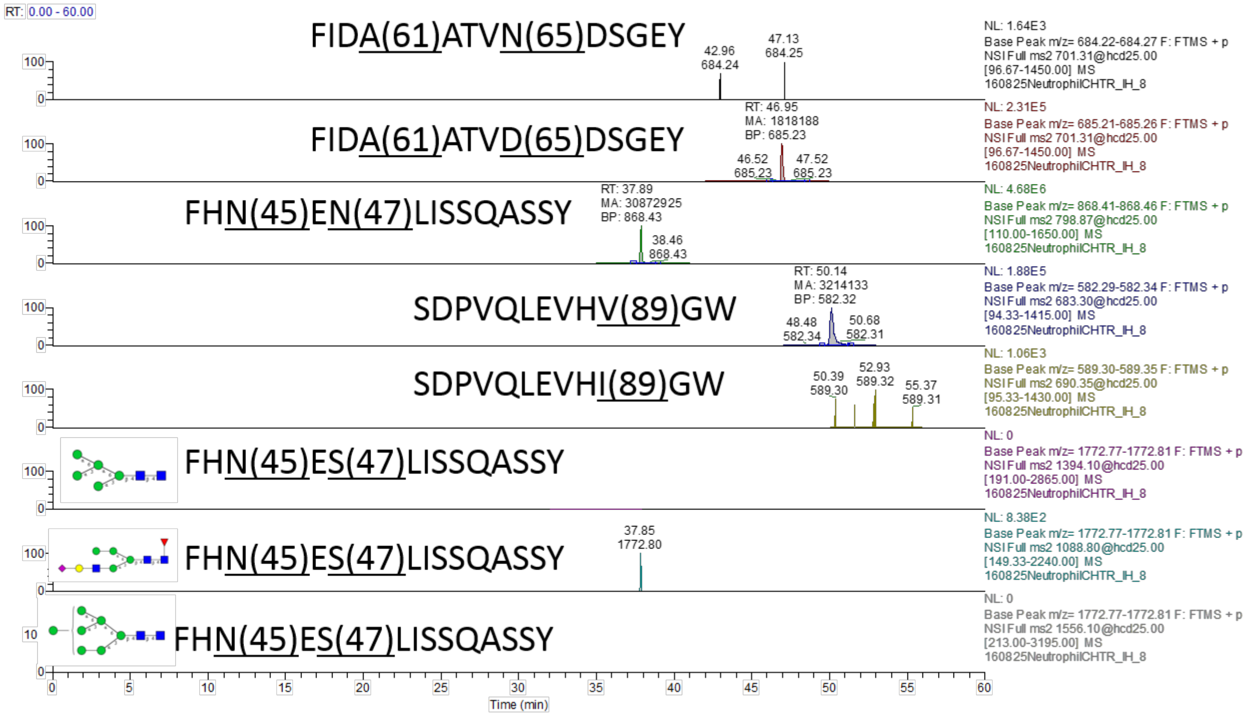

Figure S10: Example of maker peptides identified from a canonical NA1/NA1 donor. This donor has only the NA1 specific non-glycosylated N45/N47 at this site as well as the V89 and D65 marker peptides while the NA2 specific I89/N65 peptides were not detected.

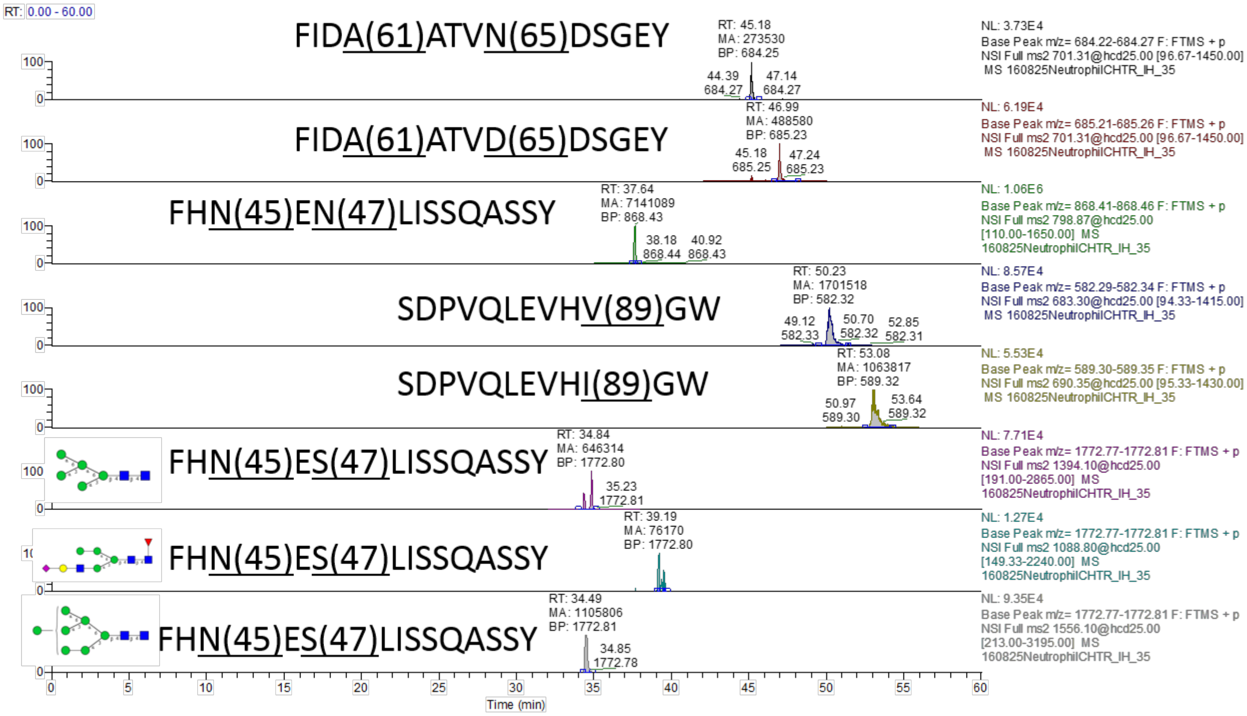

Figure S11*:* Example of maker peptides identified from a NA1/NA2 donor. This donor has marker peptides/glycopeptides for both the NA1 and the NA2 sequence at all variant residues monitored.

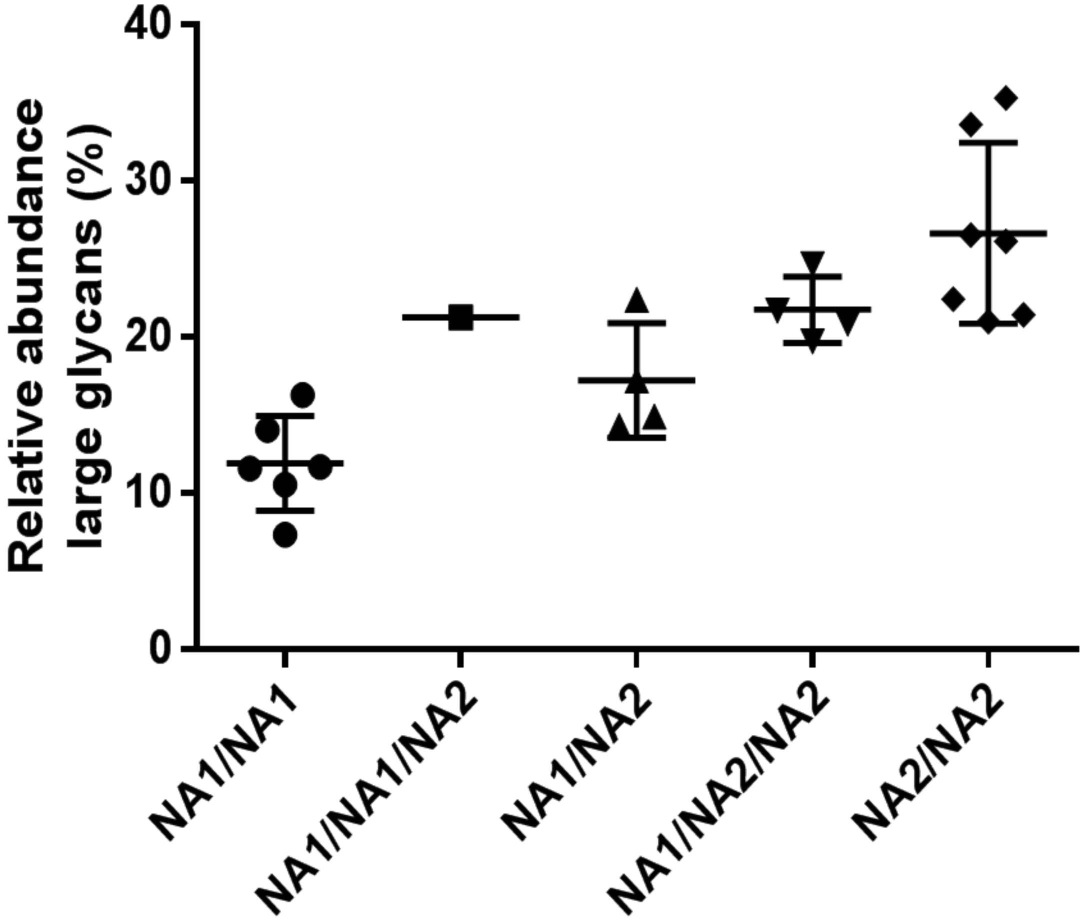

Figure S12. The relative abundance of large glycopeptides at N162 is associated with the relative copy number of the two FcγRIIIb alleles (p=0.002 one way ordinary ANOVA).

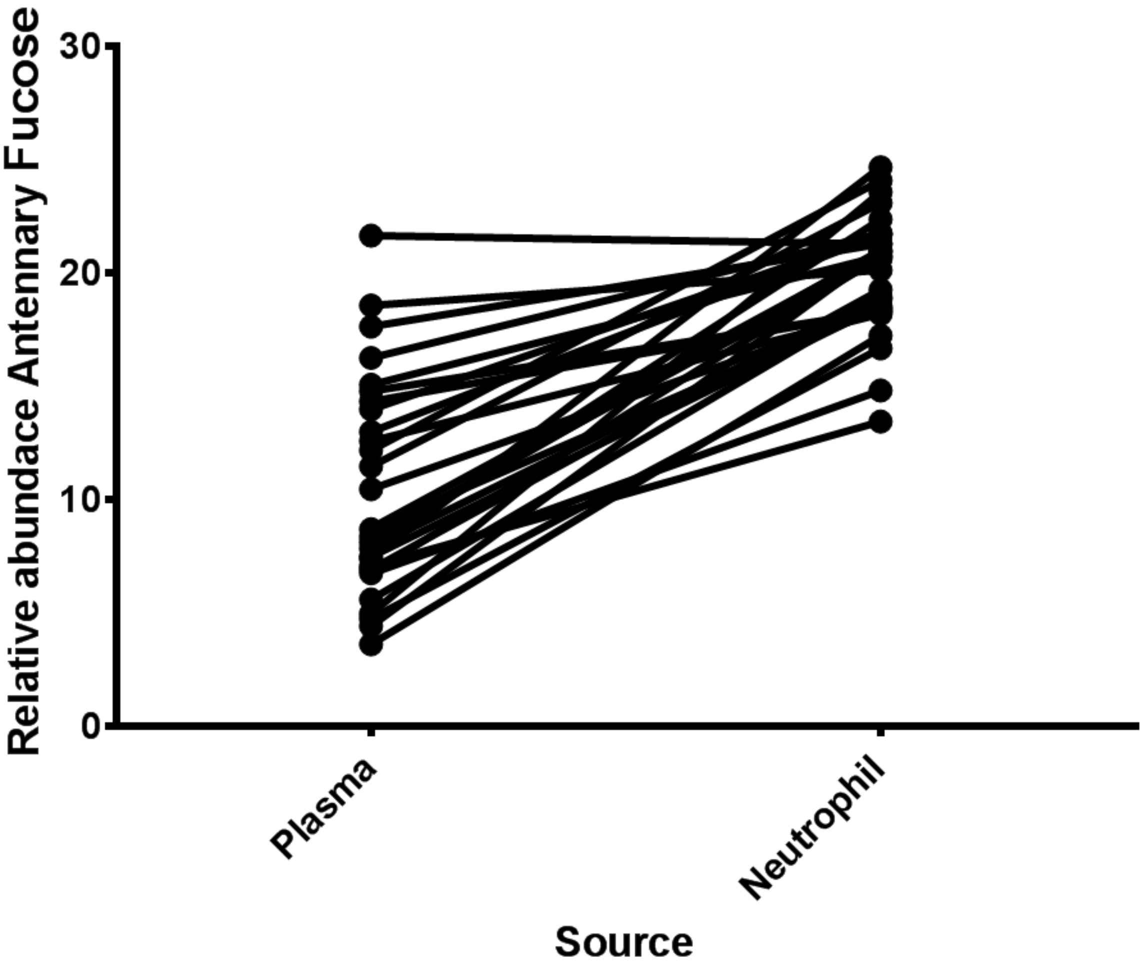

Figure S13. Comparison of the relative abundance of antennary fucose on N-glycans at N162 from neutrophils and from plasma. Relative abundance of antennary fucose containing glycopeptides at N162 are significantly higher for neutrophil FcγRIIIb compared to FcγRIIIa/FcγRIIIb from plasma ($p < 0.0001$ paired t-test). **N=30**

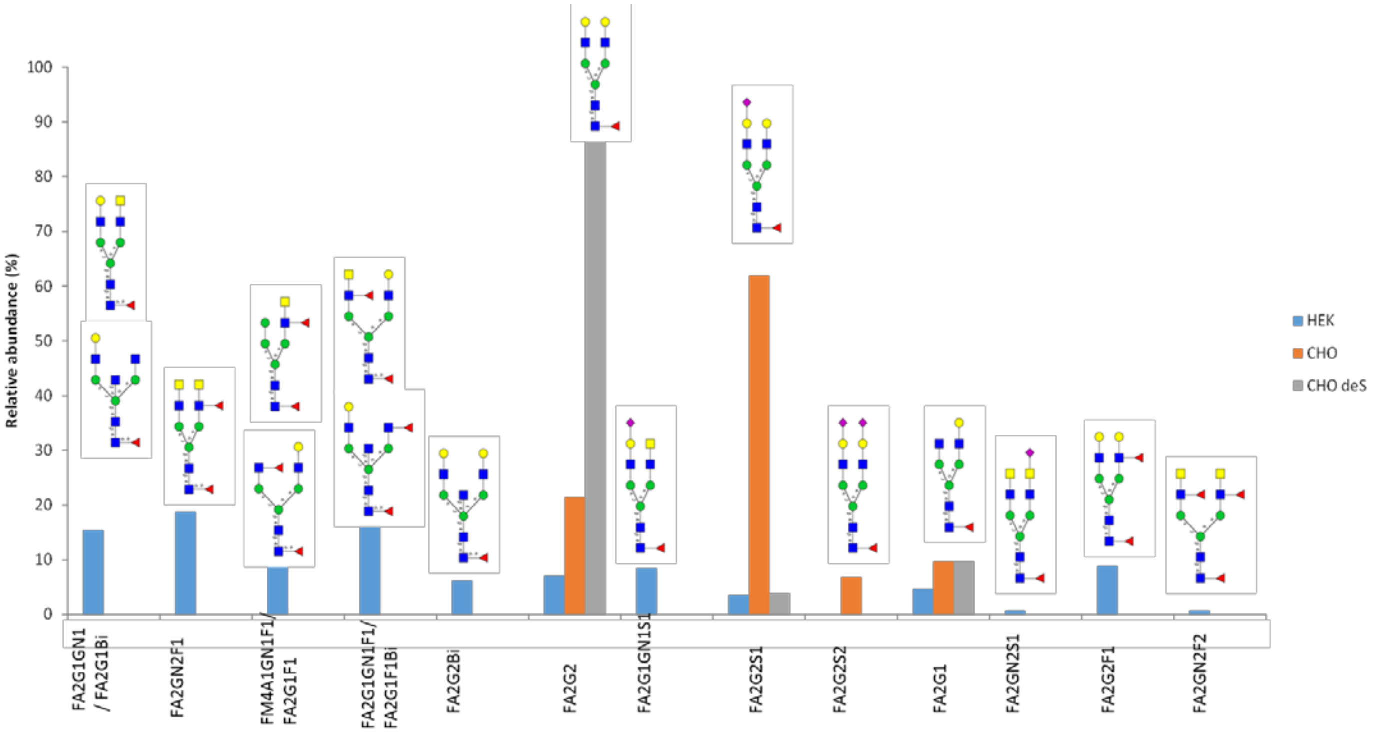

Figure S14. rhFcγRIIIa N162 glycopeptide abundance comparison between CHO 158Val, HEK 158Val and desialylated CHO 158Val. The equilibrium affinity of IgG for each of these samples was determined using SPR.

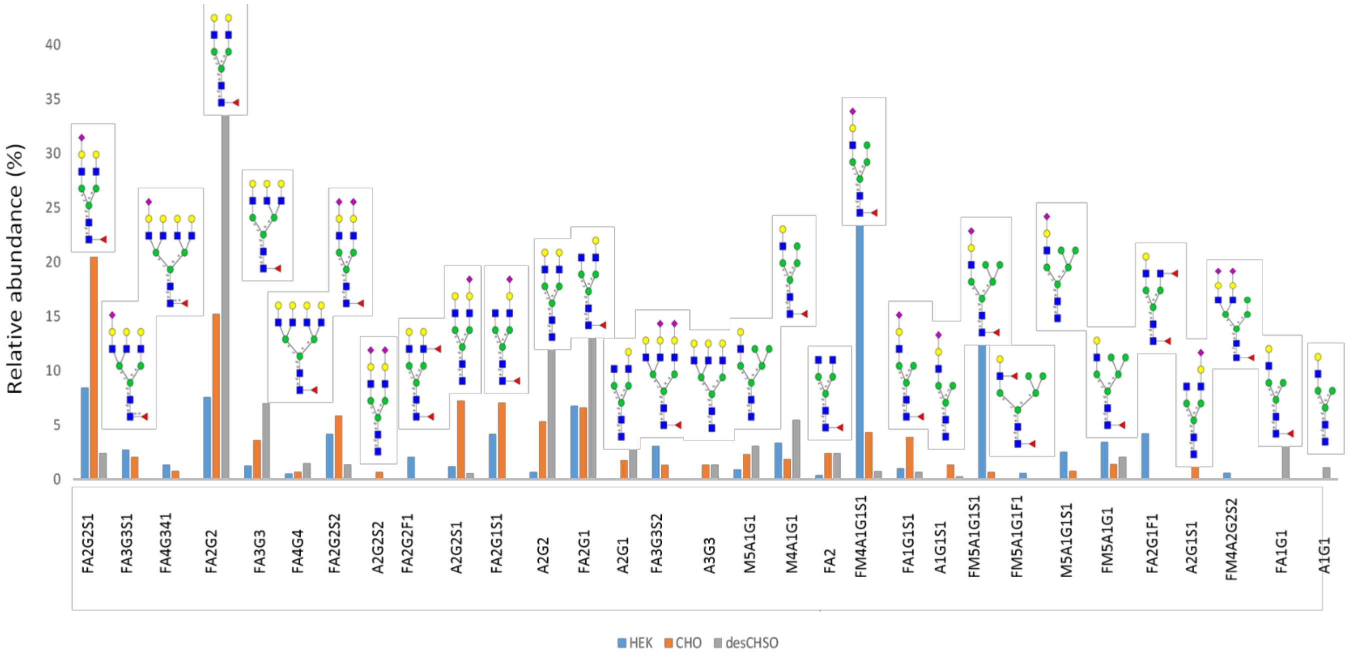

Figure S15. rhFcγRIIIa N45 glycopeptide abundance comparison between CHO 176Val, HEK 176Val and desialylated CHO 176Val. The equilibrium affinity of IgG for each of these samples was determined using SPR.

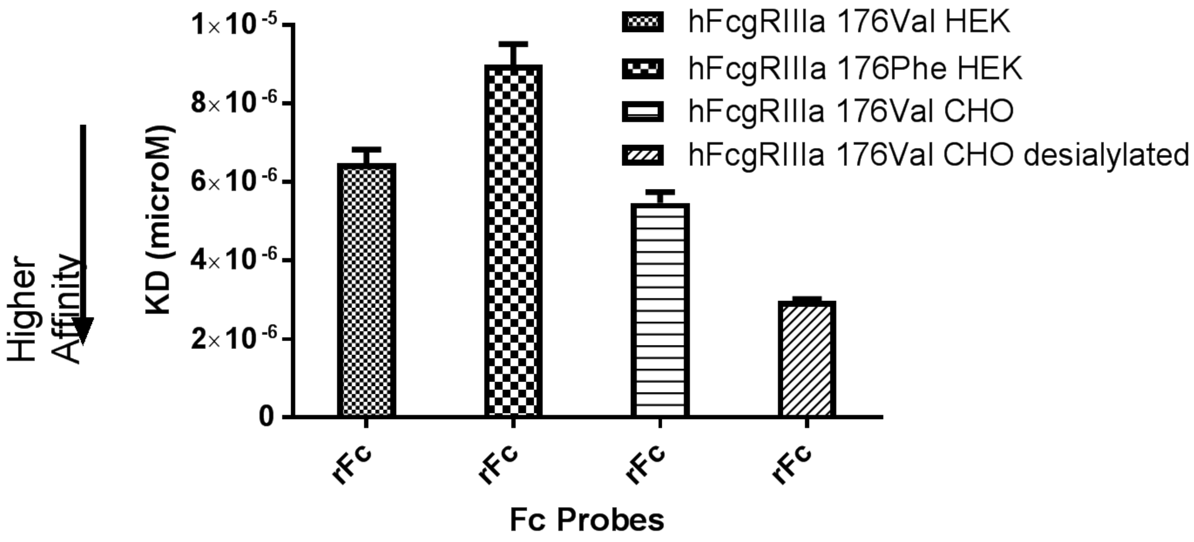

Figure S16. Equilibrium binding affinity of IgG1 Fc for rhFcγRIIIa with different glycosylation patterns either as a result of the expression system or enzymatic desialylation.

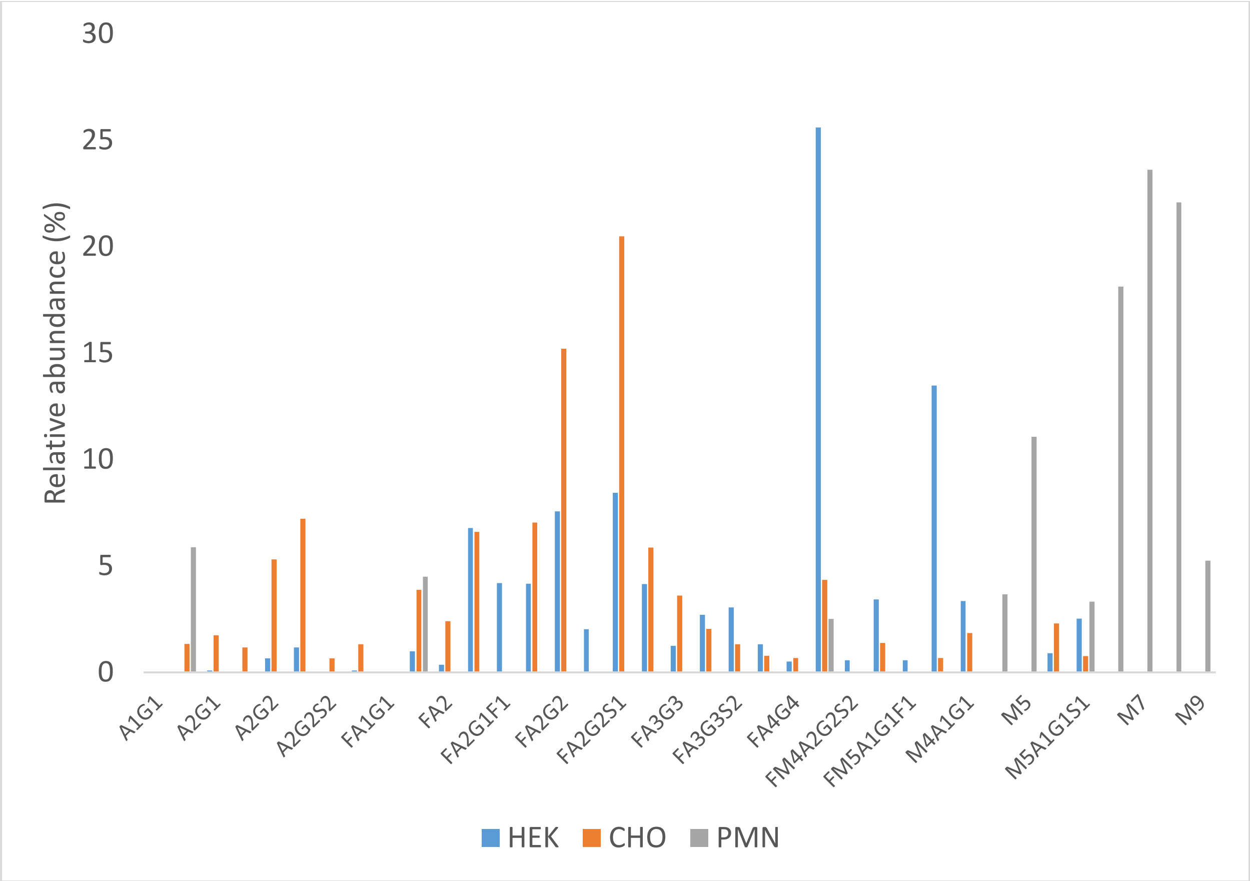

Figure S17. Comparison of FcγRIII N45 glycosylation patterns for rhFcγRIIIa expressed in HEK or CHO with hFcγRIIIb isolated from neutrophils (PMN)

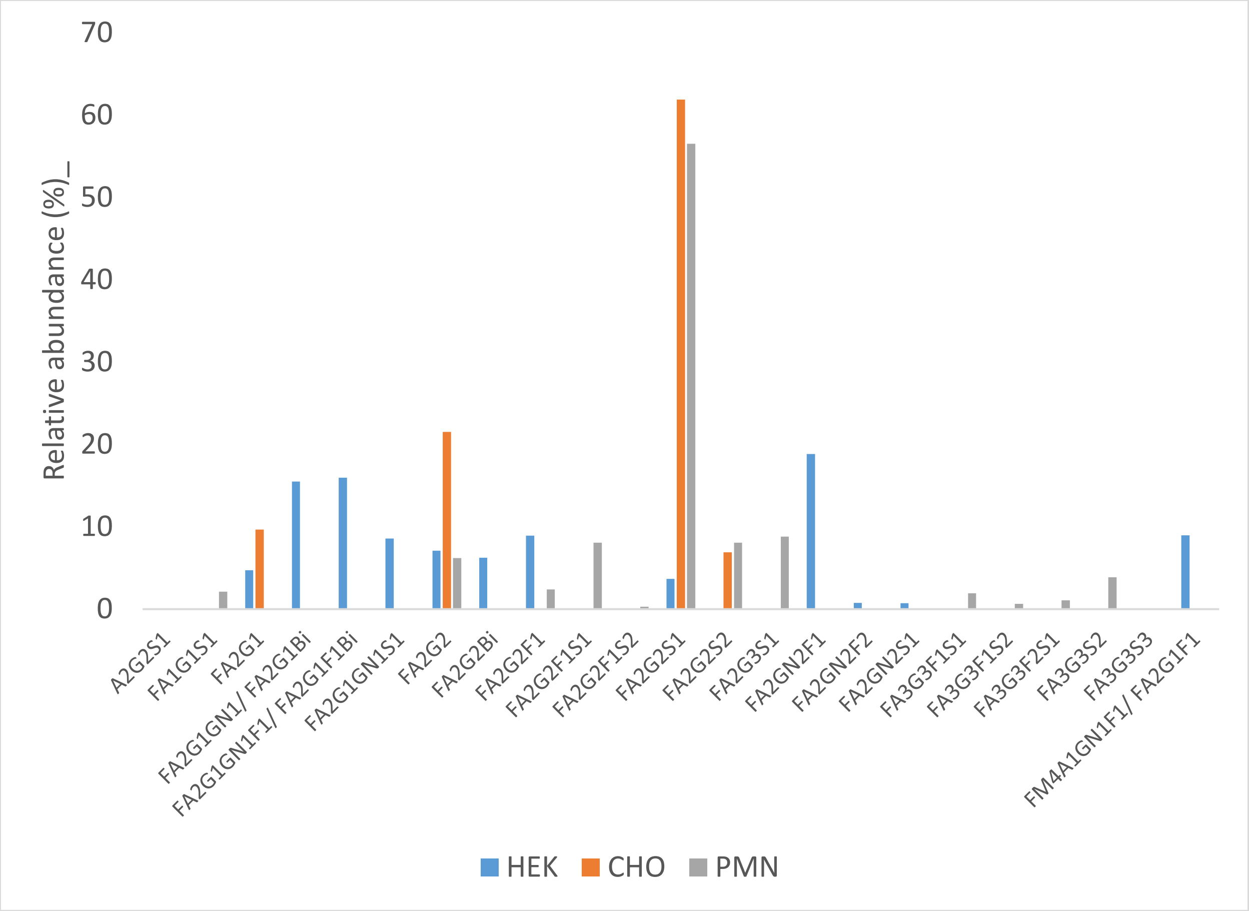

Figure S18. Comparison of FcγRIII N162 glycosylation patterns for rhFcγRIIIa expressed in HEK or CHO with hFcγRIIIb isolated from neutrophils (PMN).

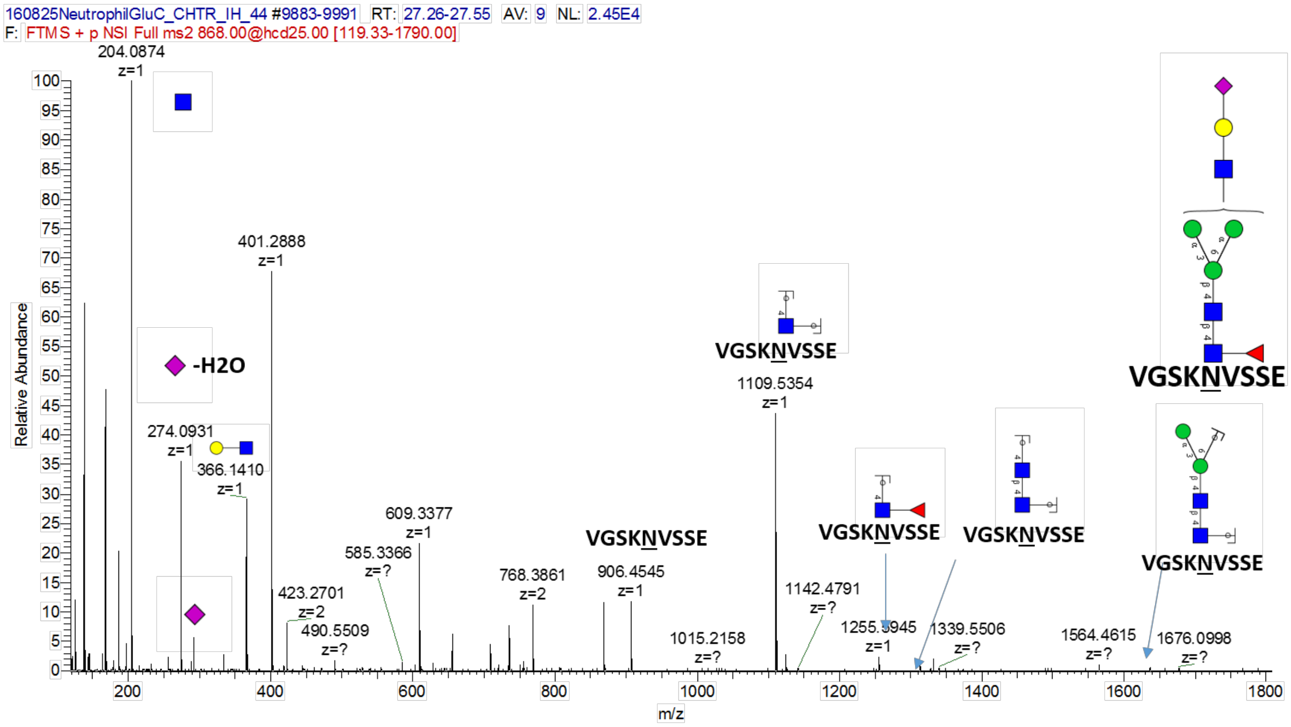

Figure s19: Annotated MS/MS spectrum for N162 FA1G1S1

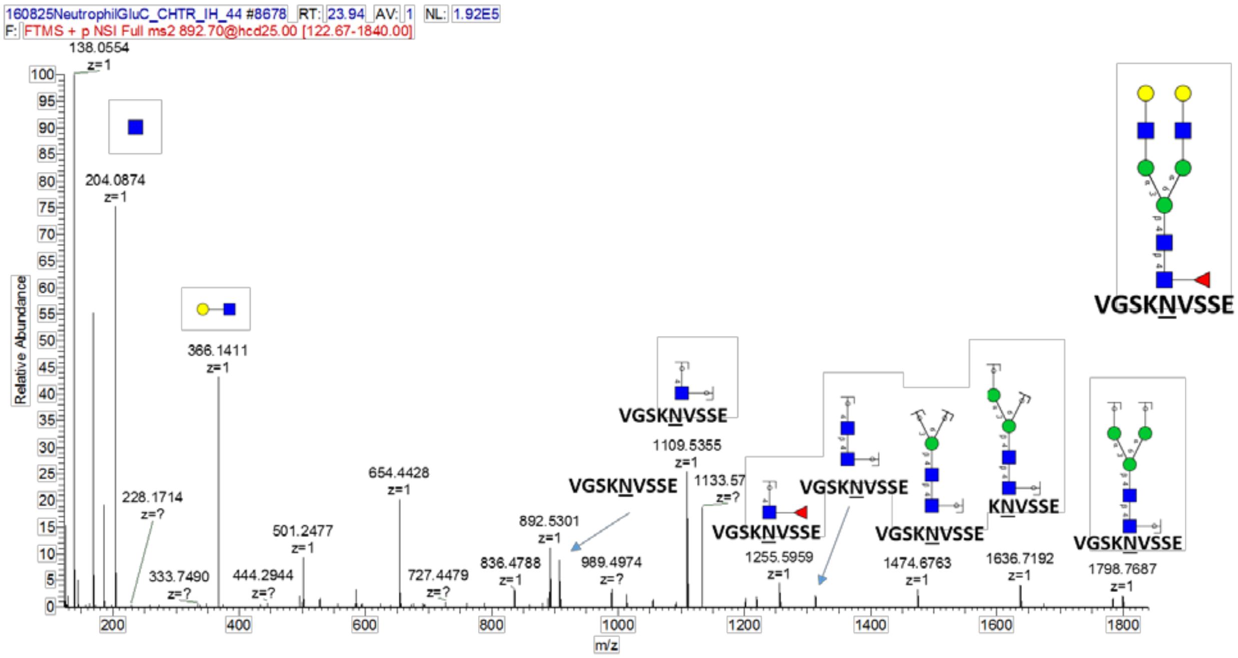

Figure s20: Annotated MS/MS spectrum for N162 FA2G2

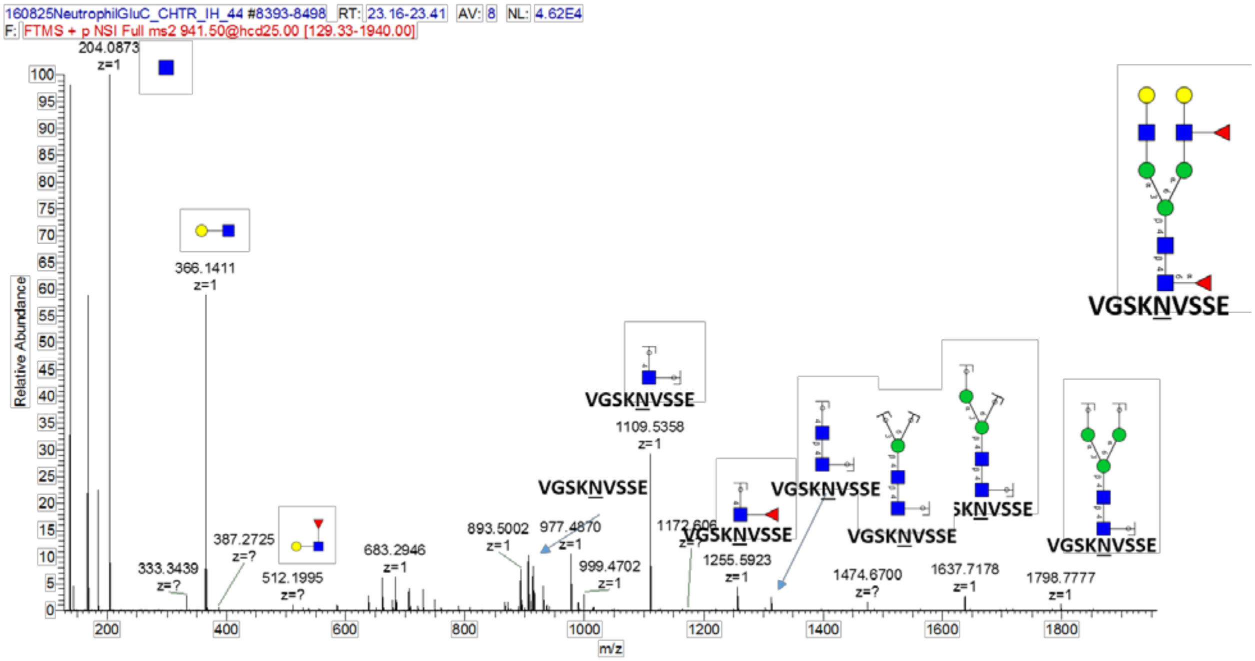

Figure s21: Annotated MS/MS spectrum for N162 FA2G2F1

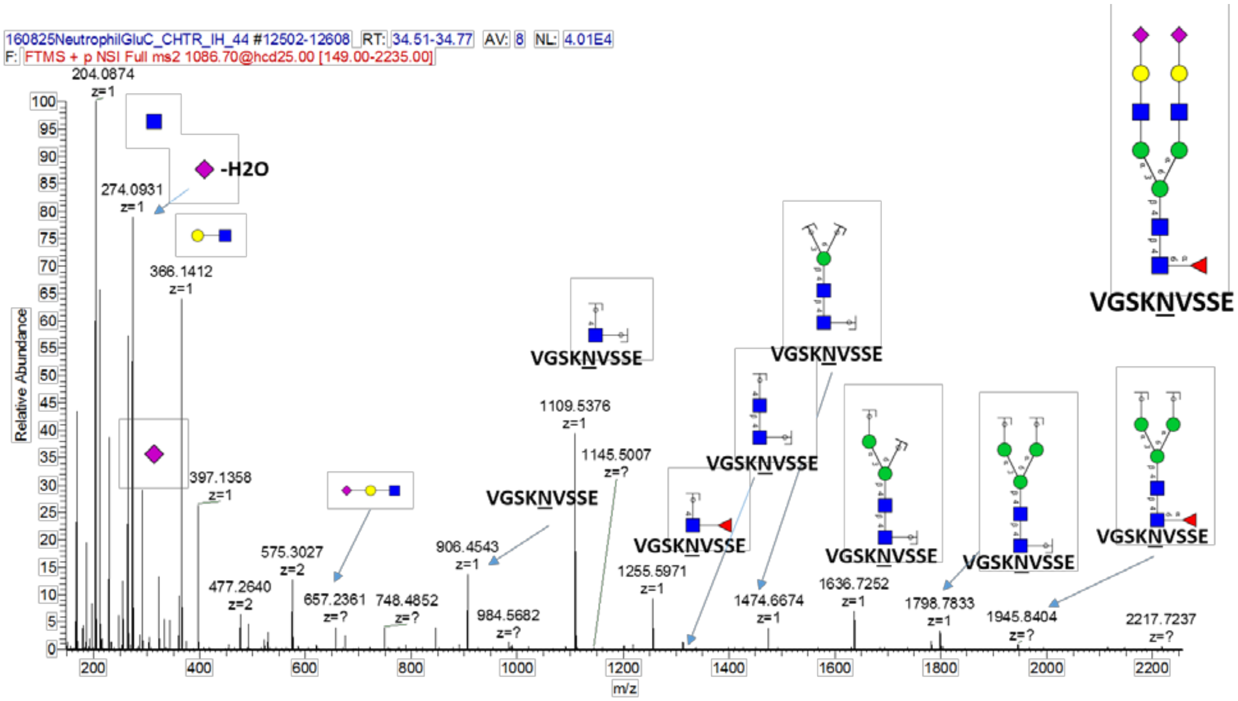

Figure s22: Annotated MS/MS spectrum for N162 FA1G2S2

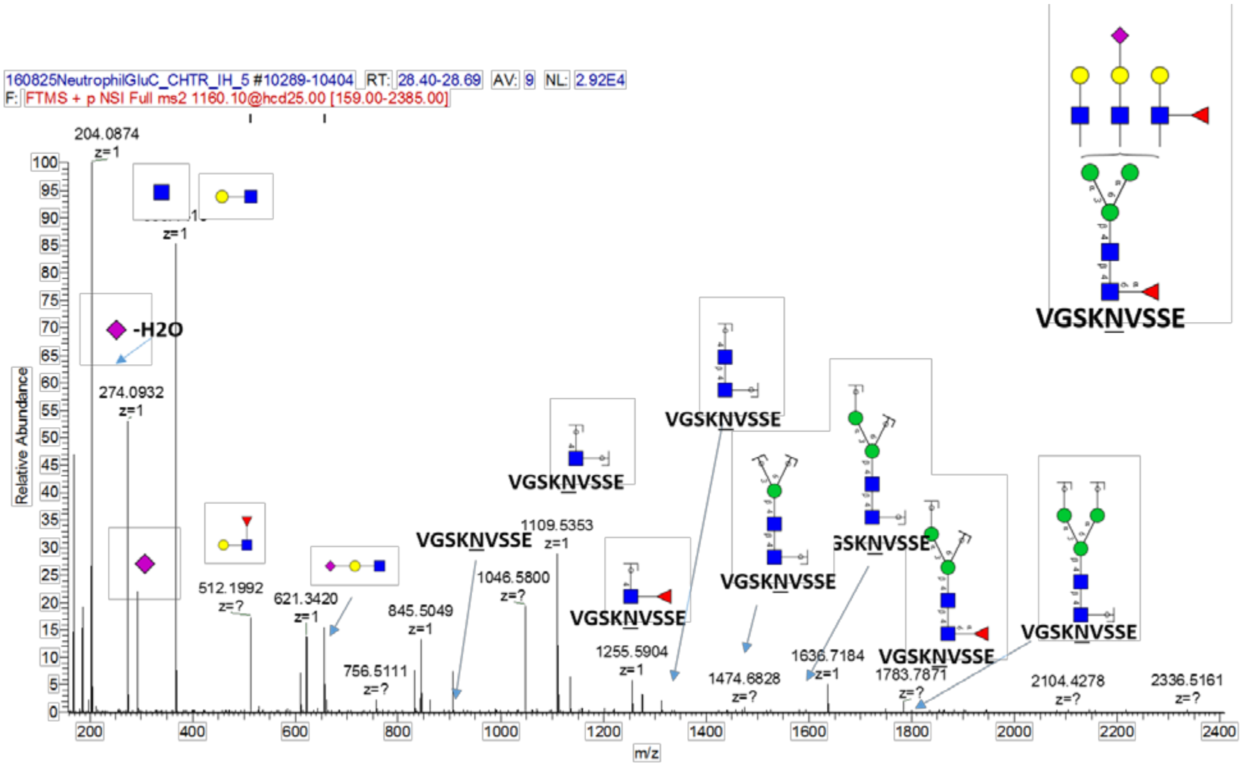

Figure s23: Annotated MS/MS spectrum for N162 FA3G3F1S1/FA2G2Lac1F1S1

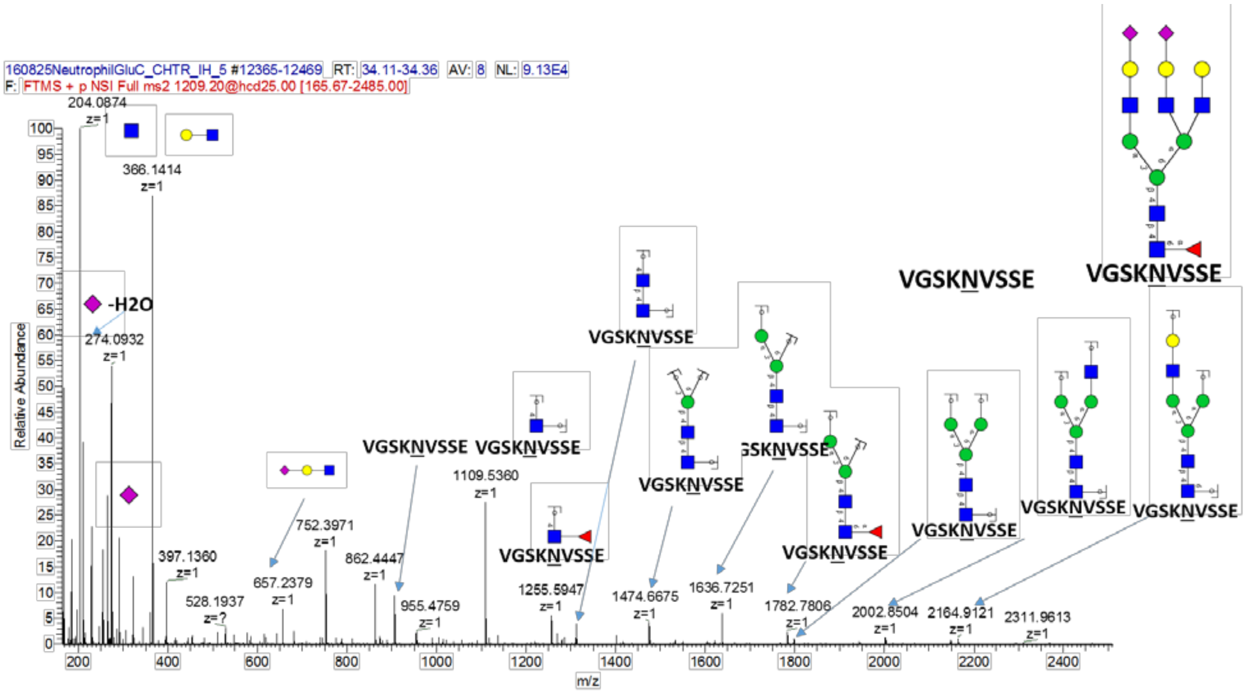

Figure s24: Annotated MS/MS spectrum for N162 FA3G3S2/FA2G2Lac1S2

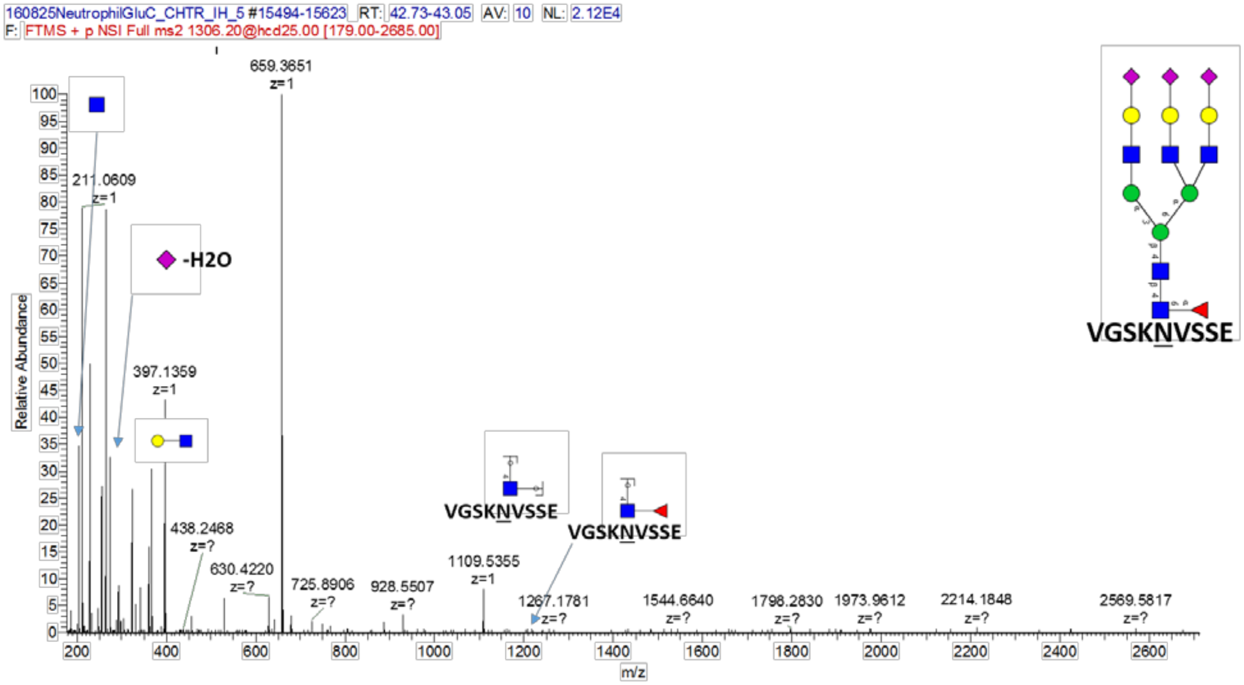

Figure s25: Annotated MS/MS spectrum for N162 FA3G3S3

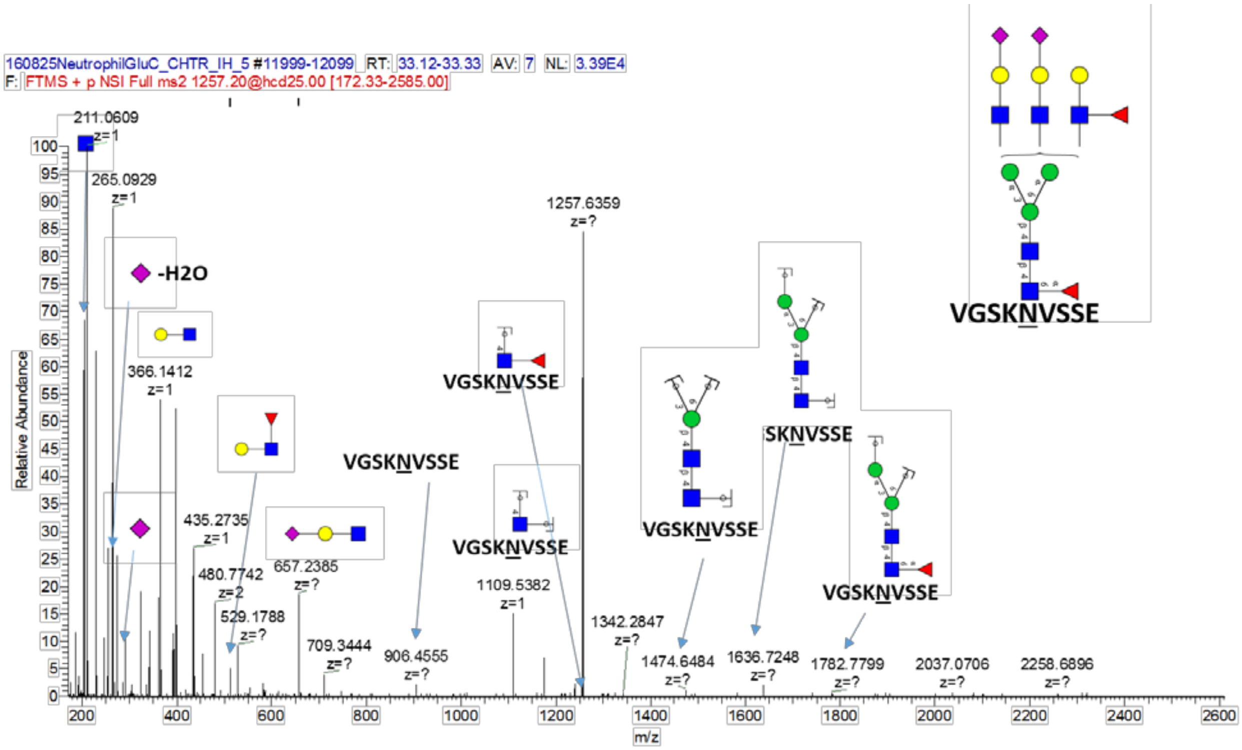

Figure s26: Annotated MS/MS spectrum for N162 FA3G3F1S1/FA2G2Lac1F1S1

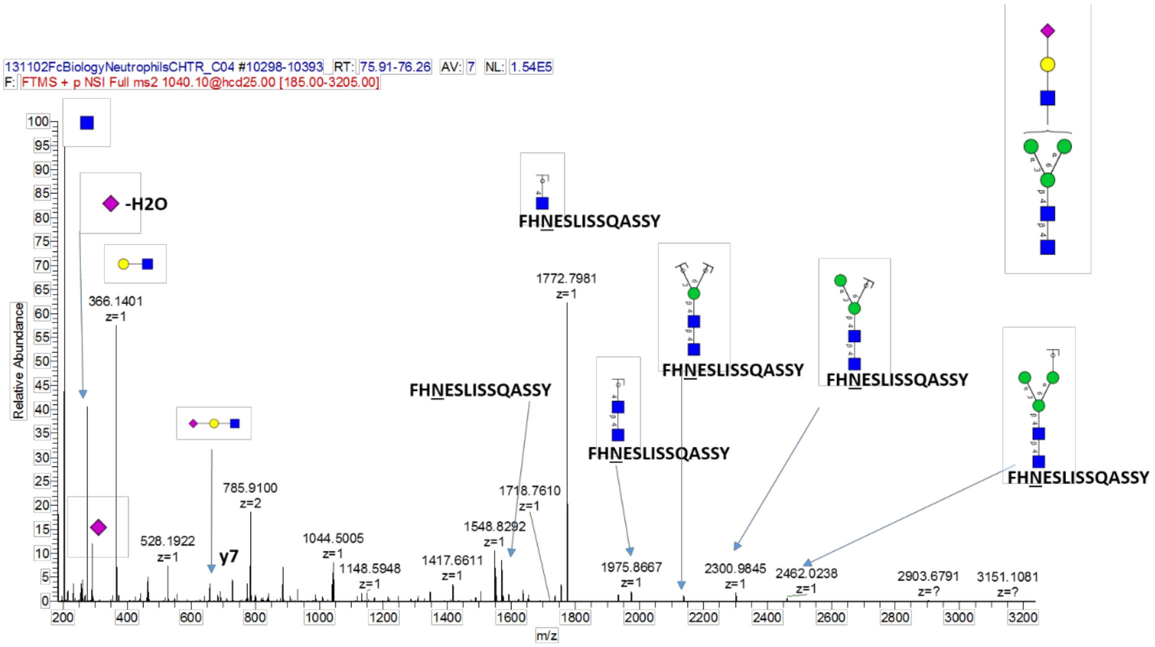

Figure s27: Annotated MS/MS spectrum for N45 A1G1S1

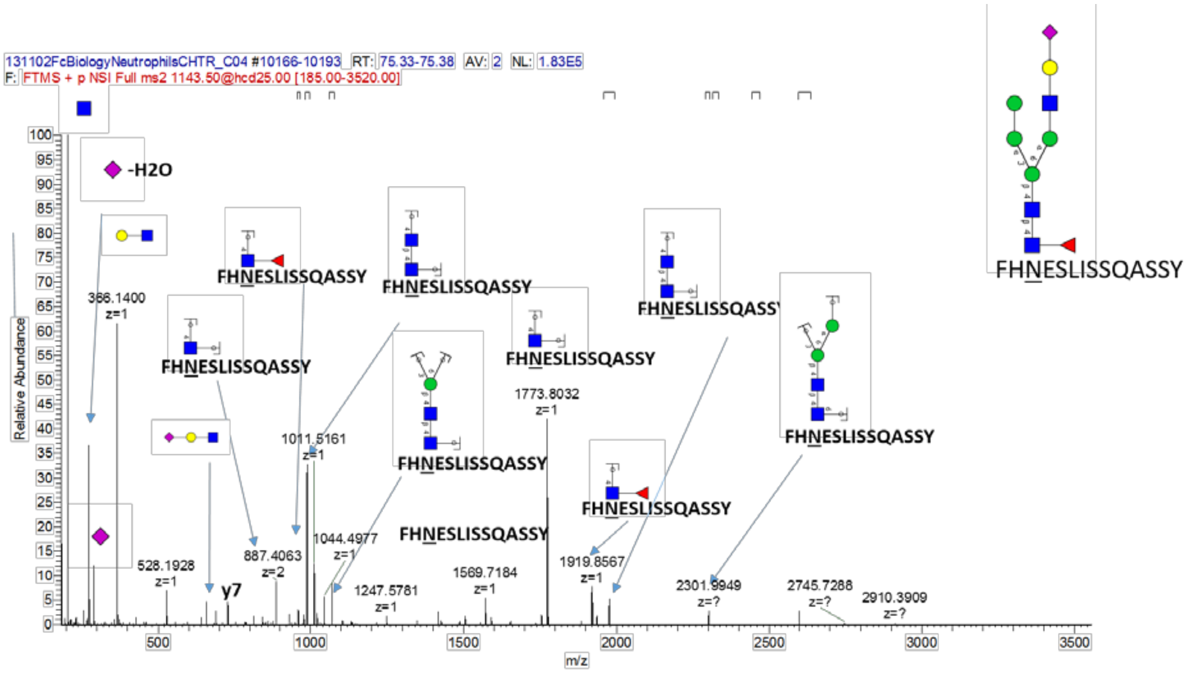

Figure s28: Annotated MS/MS spectrum for N45 FM4A1G1S1

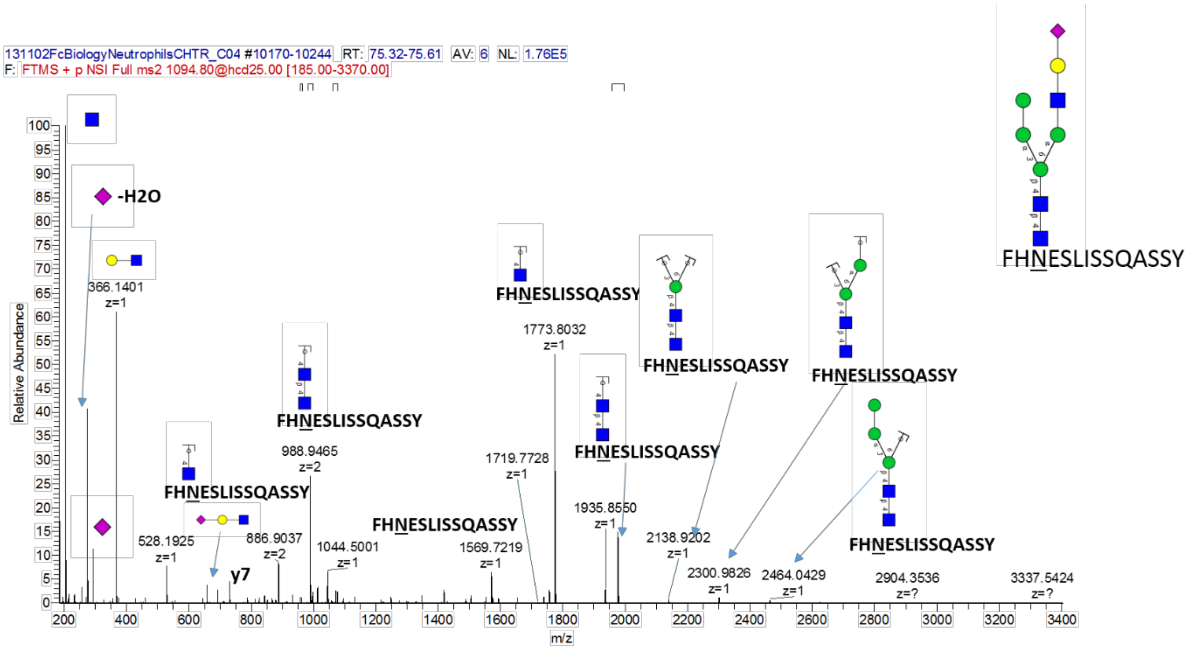

Figure s29: Annotated MS/MS spectrum for N45 M4A1G1S1

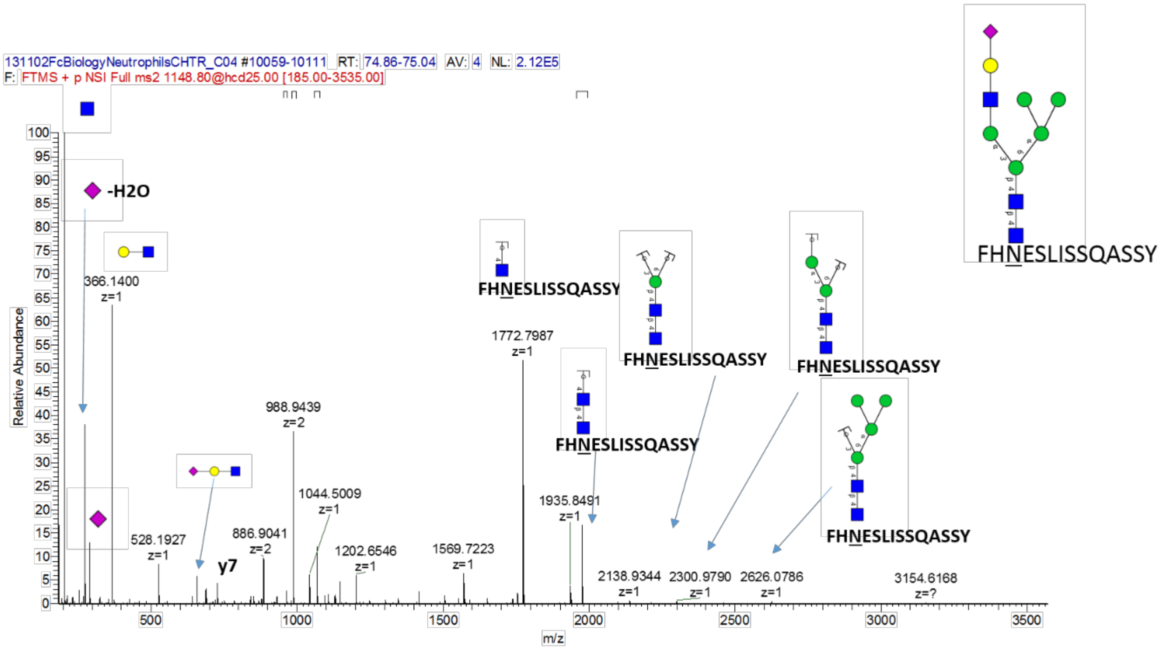

Figure s30: Annotated MS/MS spectrum for N45 M5A1G1S1

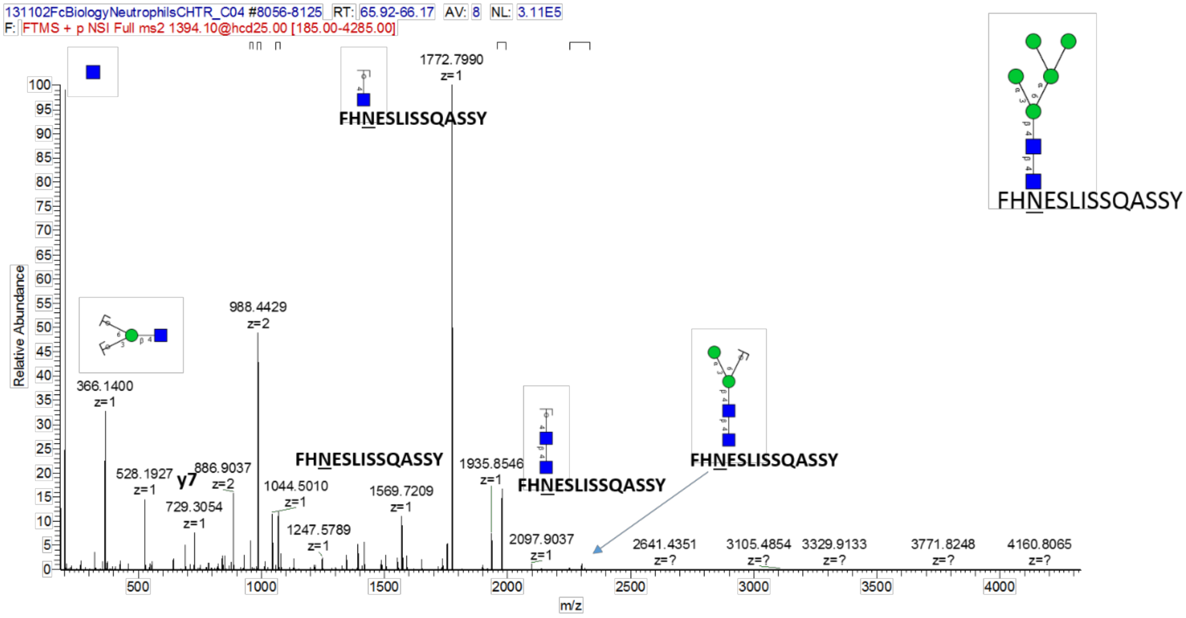

Figure s31: Annotated MS/MS spectrum for N45 M5

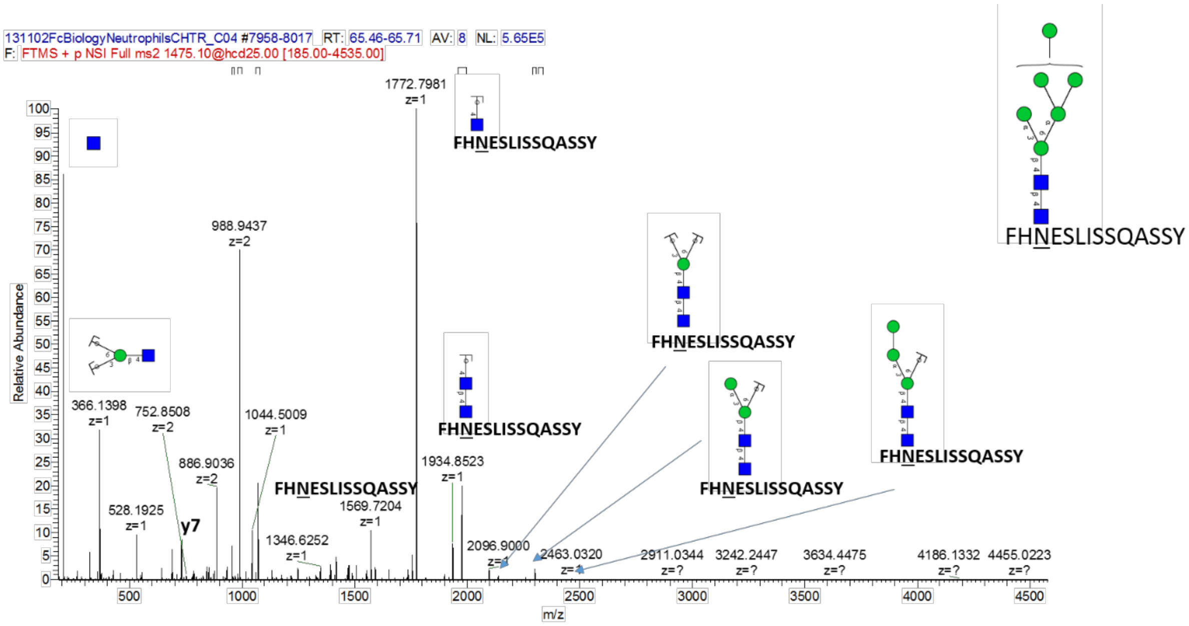

Figure s32: Annotated MS/MS spectrum for N45 M6

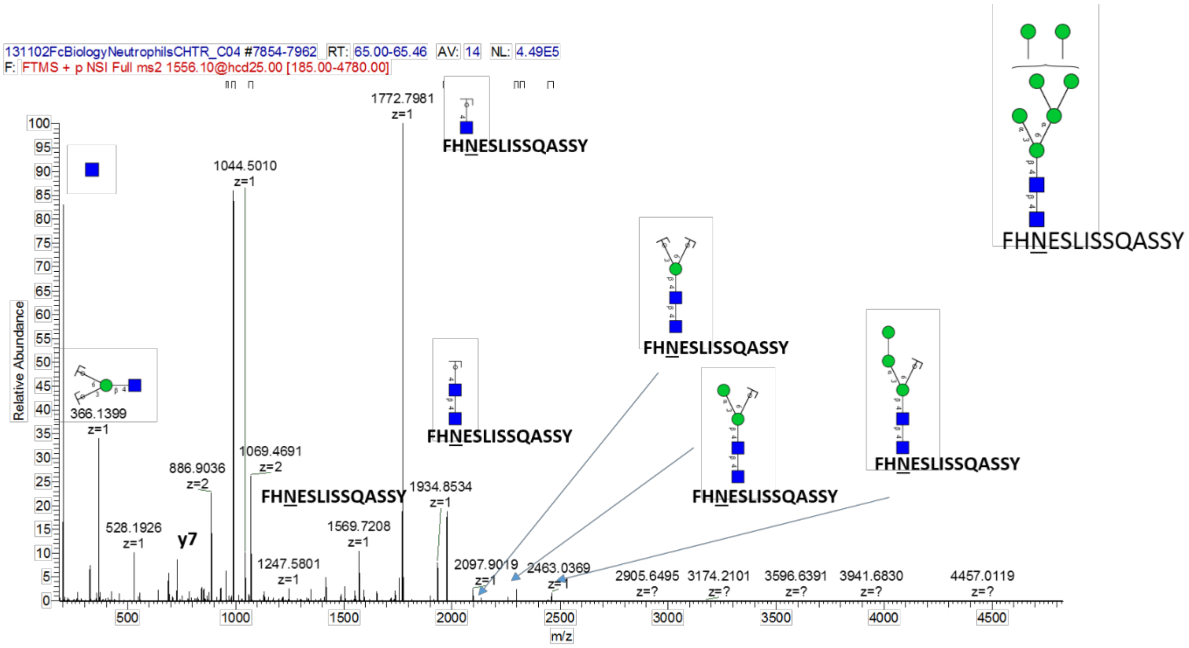

Figure s33: Annotated MS/MS spectrum for N45 M7

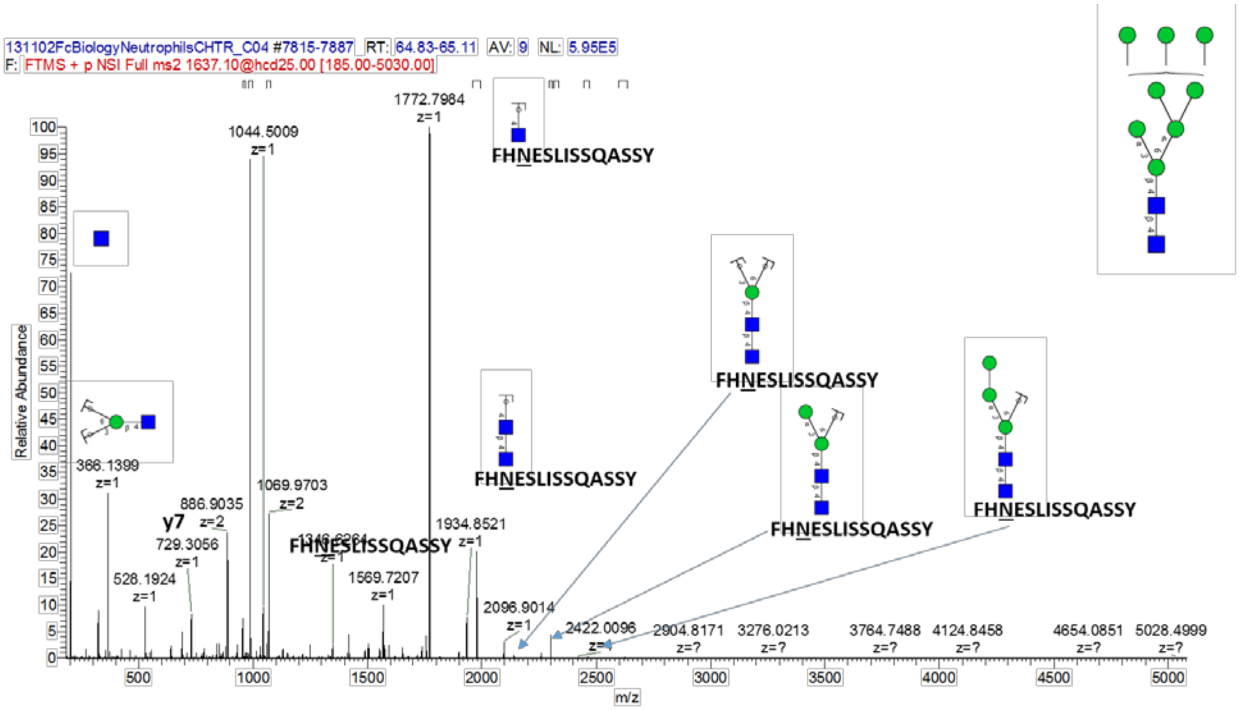

Figure s34: Annotated MS/MS spectrum for N45 M8

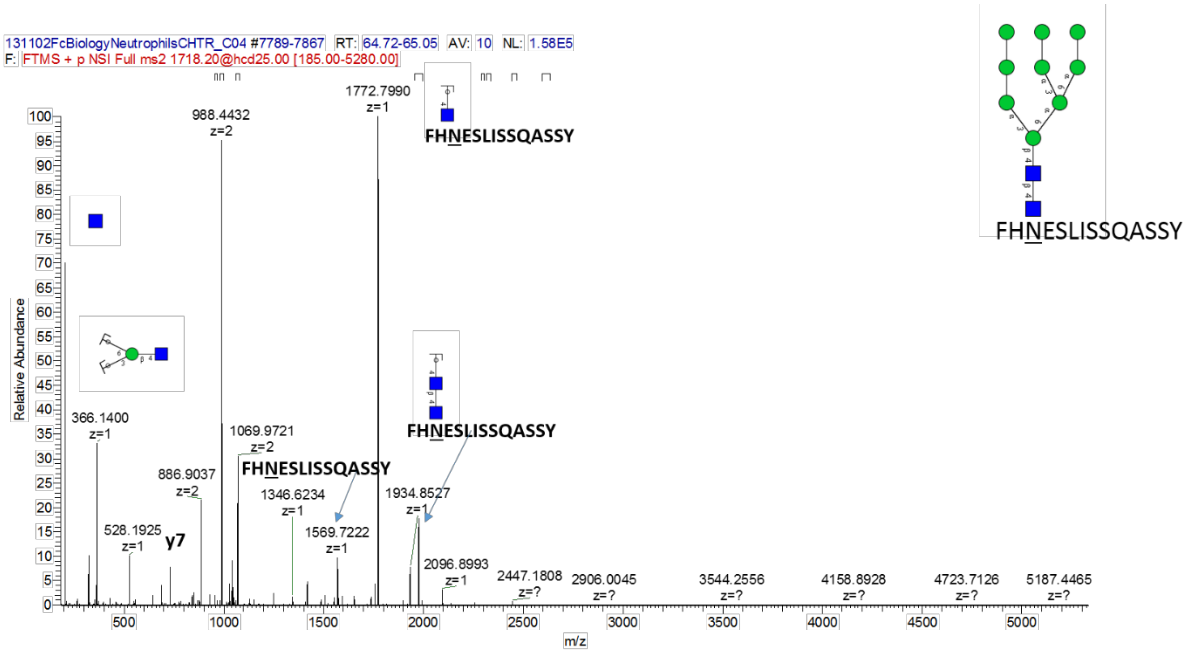

Figure s35: Annotated MS/MS spectrum for N45 M9
